# Petrological and geochemical characterization of Abu Murrat syn-orogenic I-type and post-orogenic A-type neoproterozoic granitoids, North Eastern Desert, Egypt

**DOI:** 10.1038/s41598-026-61098-1

**Published:** 2026-07-15

**Authors:** Amr El-Awady, Doaa A. Abdelnaiem, Sherif Kharbish, Beril Tanç Kaya, Amr Abdelnasser

**Affiliations:** 1 Geology Department, Faculty of Science, Zagazig University, Zagazig, 44519 Egypt; 2 Geology Department, Faculty of Science, Suez University, Suez, 43221 Egypt; 3 Geological Engineering Department, Faculty of Mines, Istanbul Technical University, Istanbul, 34469 Turkey; 4 Geology Department, Faculty of Science, Benha University, Benha, 13518 Egypt

**Keywords:** Planetary science, Solid Earth sciences

## Abstract

The Abu Murrat area, Northern Eastern Desert (NED) of Egypt, exposes two Neoproterozoic granitoid suites recording successive Pan-African evolution. The older syn-orogenic suite comprises quartz diorite, tonalite, and granodiorite, whereas the younger post-orogenic suite consists mainly of monzogranite with minor syenogranite. The syn-orogenic rocks have a moderate SiO_2_ (59.7‒69.3 wt%), high CaO (4.0‒7.3 wt%), Na_2_O (4.7‒5.7 wt%), and MgO (0.6‒2.2 wt%), and weak Eu anomalies (Eu/Eu*= ~0.8‒1). Field relations, modal QAP classification, and R1–R2, normative Ab–An–Or, A/NK–A/CNK, Ga/Al, and Nb–Y–Rb diagrams indicate calc-alkaline, magnesian, dominantly metaluminous volcanic-arc/I-type affinities. Their evolution involved assimilation and fractional crystallization (AFC), shown by decreasing MgO, Fe_2_O_3_^T^, TiO_2_, CaO, P_2_O_5_, and Sr with increasing SiO_2_, variable K/Rb and Ba/Nb, declining CaO/Na_2_O, and feldspar-controlled depletion of Eu, Sr, and Ba. The post-orogenic granites are richer in SiO_2_ (70.2‒75.8 wt%), K_2_O (5.1‒8.0 wt%), Rb (78‒178 ppm), Ga/Al, and HFSE, but poorer in CaO (0.6‒1.5 wt%) and MgO (0.04‒0.62 wt%). They also exhibit strong Eu anomalies (Eu/Eu*= ~0.05‒0.39), consistent with a within-plate A-type affinity. Zircon saturation temperatures are higher in the post-orogenic suite (~ 794–835 °C) than in the syn-orogenic granitoids (~ 635–731 °C), and monazite saturation estimates show a comparable tendency. The post-orogenic melts are interpreted as products of tonalitic lower-crustal melting during post-collisional extension, probably promoted by slab break-off and lithospheric delamination. Abu Murrat thus records the transition from subduction-related I-type arc magmatism to post-collisional A-type magmatism during Pan-African orogen collapse.

**Supplementary Information:**

The online version contains supplementary material available at 10.1038/s41598-026-61098-1.

## Introduction

Granitic rocks constitute a major component of the Arabian–Nubian Shield (ANS), representing a large proportion of both the exposed shield basement and the Egyptian Eastern Desert basement^1^. The ANS formed during the Neoproterozoic Pan-African Orogeny, broadly between ca. 900–550 Ma, through oceanic-arc generation, ophiolite emplacement, terrane accretion, basin closure, and final Gondwana assembly^2,3^. Its tectono-magmatic evolution is commonly divided into overlapping pre-collisional, syn-collisional, and post-collisional stages^4^. The pre-collisional stage involved intra-oceanic arc development and ophiolite obduction at ca. 800–750 Ma ^5–8^; the syn-collisional stage, ca.720–630 Ma, was marked by arc assemblages and calc-alkaline plutonism^9^; and the late- to post-collisional stage, beginning at ca. 620–580 Ma, was associated with crustal thickening, extensional collapse, and widespread calc-alkaline to alkaline intraplate magmatism^10,11^. These processes are expressed in the Egyptian Eastern Desert by ophiolitic mélanges, arc-related metavolcanic–metasedimentary successions, calc-alkaline syn- to late-orogenic plutons, post-collisional mafic–felsic intrusions, and late shear systems partly associated with Najd-style strike-slip deformation^2,11,12^. This regional framework is critical for interpreting the Abu Murrat granitoids, whose mineralogical and geochemical features record successive stages of hydrous arc magmatism, crustal thickening, post-collisional extension, and mantle-assisted lower-crustal melting. The tectonomagmatic evolution of the northern ANS is broadly established. However, the locality-scale mechanism linking subduction-related magnesian I-type arc magmatism to post-orogenic ferroan A-type magmatism remains poorly constrained, especially where older and younger intrusive suites occur in close spatial association.

In the Egyptian Eastern Desert, Neoproterozoic granitoids are commonly classified into two groups^13,14^: (1) syn-orogenic “Older Granites” (grey granitoids), emplaced during the orogenic collision stage (~ 850–614 Ma)^15–18^; and (2) post-orogenic “Younger Granites” (pink granitoids), emplaced in the post-collisional extensional stage (~ 610–550 Ma)^18–22^. The Older Granites are typically calc-alkaline I-type bodies ranging from quartz diorite to granodiorite^16–18^, often deformed and emplaced during active subduction and collision^2,14,23^. By contrast, the Younger Granites are largely undeformed, highly evolved granites (commonly metaluminous to mildly peraluminous A-type) that crystallized after collision under extensional, within-plate conditions. They include monzogranites, syenogranites, and alkali-feldspar granites, generally without reaching extreme peralkaline compositions^15,24,25^. Most previous studies have addressed these granitoid suites at a regional scale or mainly through whole-rock geochemistry. Consequently, integrated studies that combine field relationships, modal petrography, mineral chemistry, and whole-rock data from a single locality remain important. Such studies help to evaluate whether paired granitoid suites record a coherent transition from convergent-margin magmatism to post-collisional extensional settings.

The Abu Murrat area, located near Wadi Baroud southwest of Safaga in the Northern Eastern Desert (NED), Egypt, provides an excellent natural setting for examining late Neoproterozoic granitoid evolution. Syn-orogenic granitoids, post-orogenic gabbros, post-orogenic granites, enclaves, dike swarms, and cross-cutting intrusive relationships are all exposed within a relatively restricted area^26–29^. Although the transition from subduction-related I-type magmatism to post-collisional A-type magmatism is well documented in the northern Arabian–Nubian Shield, the Abu Murrat assemblage allows this transition to be examined within a single structural and magmatic framework rather than through isolated plutons. Previous studies, particularly those of Awad, et al. ^27^ and El-Awady, et al. ^26^, focused mainly on Abu Murrat gabbroic rocks and granitoids from nearby Eastern Desert localities. In contrast, the syn-orogenic and post-orogenic granitoid suites of Abu Murrat have not been investigated together in a comprehensive petrological and geochemical framework. Therefore, this contribution does not seek to redefine the regional tectonic evolution of the ANS, but rather to refine the petrogenetic interpretation of the Abu Murrat granitoids and assess how this area records the shift from convergent-margin to post-collisional extensional magmatism. The novelty of this study lies in integrating field relations, petrography, mineral chemistry, whole-rock geochemistry, and thermal estimates to addresses four linked questions: (1) What petrographic, mineral-chemical, and geochemical criteria distinguish the syn-orogenic and post-orogenic granitoids at Abu Murrat area; (2) Do the post-orogenic monzogranites satisfy the mineralogical and geochemical criteria for A-type granite affinity; (3) What evidence constrains the relative roles of fractional crystallization, crustal assimilation, and lower-crustal melting during the magma evolution; and (4) What thermal regime and tectonic transition are recorded by the Abu Murrat granitoids during the late Neoproterozoic evolution of the northern Arabian–Nubian Shield. In addressing these questions, we evaluate how the Abu Murrat granitoid assemblage refines local evidence for the transition from subduction-related I-type magmatism to post-orogenic A-type magmatism in the NED of Egypt. The temporal framework adopted here is based on regional geochronological constraints and field relationships, as no new isotopic ages are presented in this contribution.

### Geological setting

**Fig. 1:**
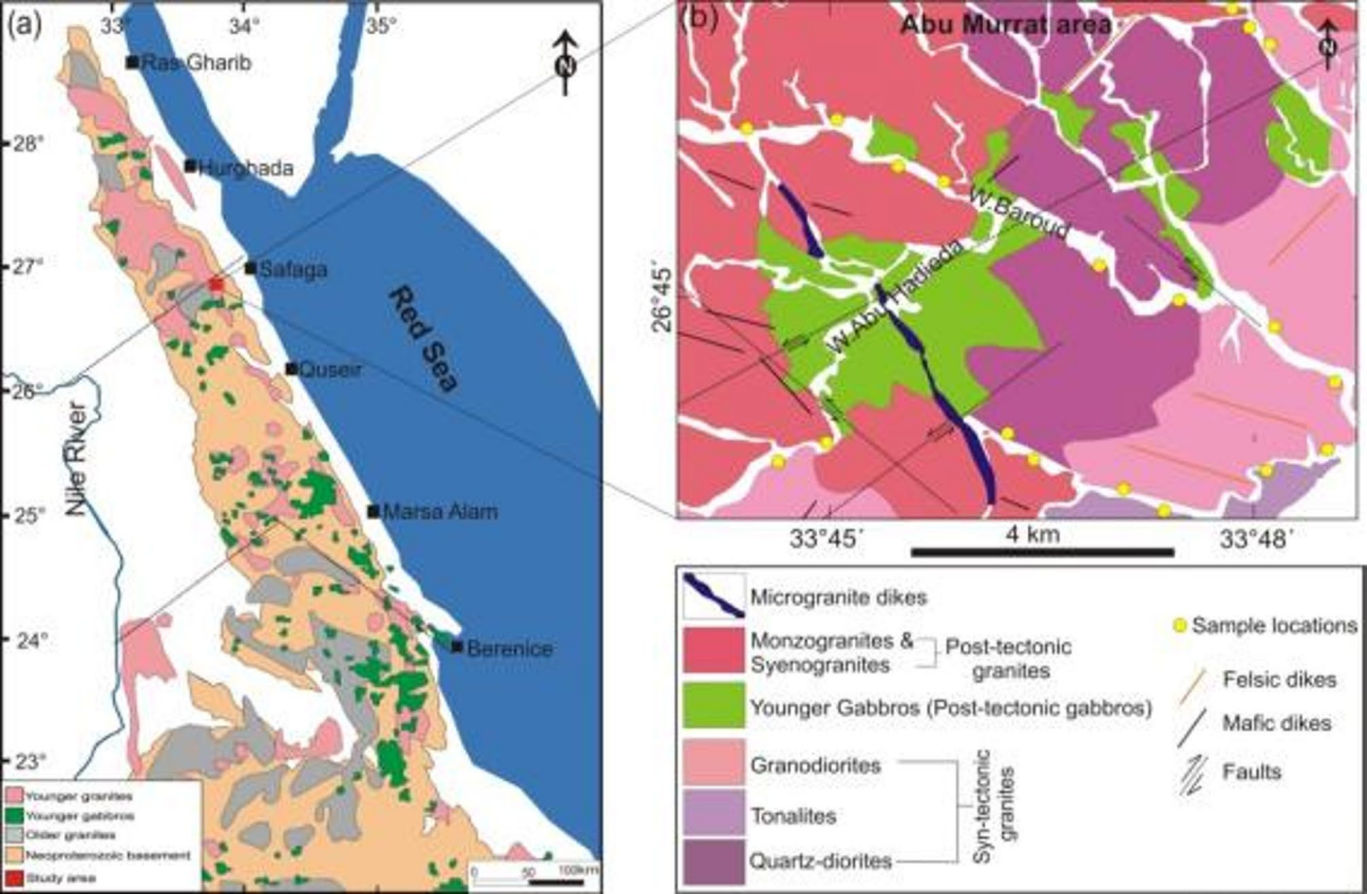
(**a**) Simplified map of Neoproterozoic syn-orogenic granite, post-orogenic gabbro, and post-orogenic granite distribution in Egyptian Eastern Desert, showing the boundaries between the Northern Eastern Desert (NED), Central Eastern Desert (CED) and Southern Eastern Desert (SED). The Abu Murrat study area is marked by a red square near Safaga, close to the NED–CED boundary. (**b**) Geological map of the Abu Murrat area, NED, modified after Awad, et al.^27^ and El-Awady, et al.^26^. The map was compiled and graphically edited using CorelDraw Graphics suite X3 (Corel Corporation. Ottawa, Canada; https://www.coreldraw.com).

The Abu Murrat area lies in the northern part of the Eastern Desert of Egypt, approximately 25 km southwest of Safaga (Fig. 1). It is situated close to the boundary between the Northern and Central Eastern Desert and is intersected by major NW–SE (Najd) and NE–SW (Barramiya-Mubarak) strike-slip fault systems^26–30^. The mapped rock units in the area occur in a chronological sequence and include syn-orogenic granites, post-orogenic gabbros, post-orogenic granites, and late felsic and mafic dikes (Fig. 1)^26–28^.

**Fig. 2:**
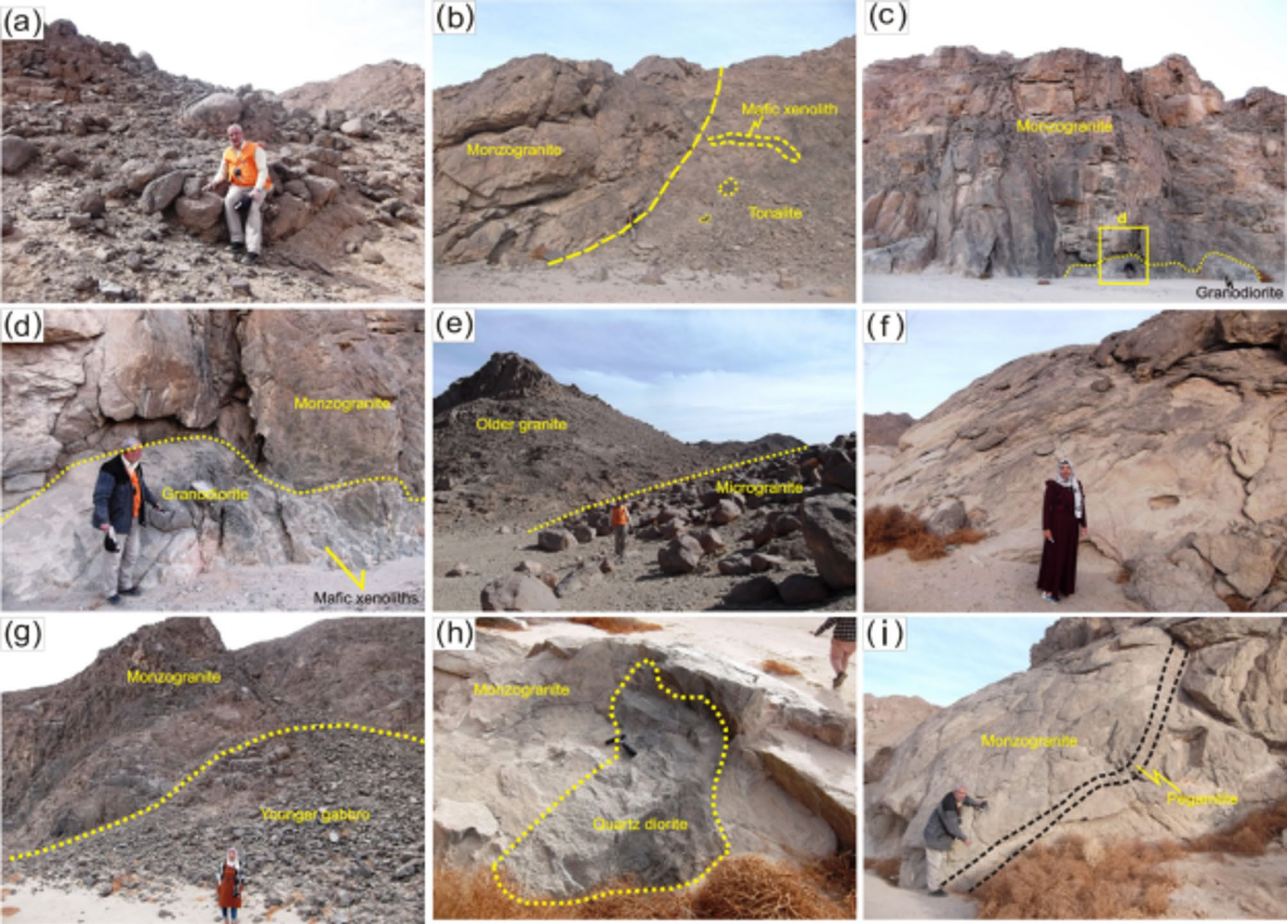
Field photographs of Abu Murrat granitoids. (**a**) Spheroidally weathered surface of syn-orogenic granite (tonalite). (**b**) Sharp intrusive contact between post-orogenic granite (monzogranite) and syn-orogenic granite (granodiorite; hosting dark mafic xenoliths). (**c**) Post-orogenic granite sheet overlying syn-orogenic granite (granodiorite) and incorporating a large xenolith of the older rock. (**d**) Close-up of intrusive contact of monzogranite against granodiorite of the syn-orogenic granite, with mafic xenoliths in the latter near the contact. (**e**) Deformed NE–NNE-trending microgranite dike intruding syn-orogenic granite, offset by a NW–SE fault. (**f**) Exfoliated and tafoni-bearing weathering surface of post-orogenic granite. (**g**) Sharp intrusive contact between post-orogenic granite and a body of post-orogenic gabbro. (**h**) Quartz diorite enclave of syn-orogenic granite hosted within monzogranite. (**i**) Pegmatite vein cutting monzogranite.

The syn-orogenic granites are the most extensively exposed unit in the area. They form low to moderate relief hills and locally display weak, non-pervasive gneissose fabric, interpreted as syn-orogenic ductile deformation rather than primary magmatic or later metamorphic foliation. They are gray to greenish-gray in color and display intense chemical weathering features such as fracturing, spheroidal weathering, and exfoliation (Fig. 2a). Compositionally, these granitoids range from quartz diorite (notably in the central area) to tonalite and granodiorite (occurring toward the east and southeast). They have sharp intrusive contacts with the post-orogenic granite (monzogranite) suite (Fig. 2b–d) and are cut by numerous dikes, including felsic microgranite dikes and occasional mafic dikes (Fig. 2e). They also host abundant enclaves of darker, mafic to intermediate composition (microdioritic enclaves), especially near contacts with the monzogranites (Fig. 2b, d).

The post-orogenic granite suite forms the highest and most prominent topographic features in the region, often capping steep hills. These rocks are typically pink to red, medium- to coarse-grained granites composed predominantly of monzogranite with minor syenogranite phases. They are generally more resistant to erosion than the syn-orogenic granites (Fig. 2b–d) and show only mild weathering (e.g., isolated spheroidal boulders, tafoni cavities, and surface exfoliation; Fig. 2f). The post-orogenic granites intrude and locally overlie the syn-granites and the post-orogenic gabbros along sharp, discordant contacts (Fig. 2b–d, g). They occasionally contain xenoliths of the older quartz diorite (Fig. 2h). Aplite, microgranite, and pegmatite veins frequently traverse the post-orogenic granites (Fig. 2i). Narrow chilled margins only a few centimeters thick can be observed at some post-orogenic granite contacts, indicating rapid cooling against the older host rocks. These post-orogenic granites are cut by an orthogonal joint system (generally E–W and N–S), reflecting regional fault-related joint patterns.

Late microgranite dikes in the area occur within narrow shear zones and also intrude along pre-existing structural trends (Fig. 2e). These dikes are often strongly altered (with hematitic staining) and enriched in U and Th due to hydrothermal activity, especially along fractures^31^. The microgranite dikes strike NE–NNE, parallel to the local shear fabric, and are offset by later NW–SE faults (Fig. 2e).

### Petrographic characteristics

**Fig. 3:**
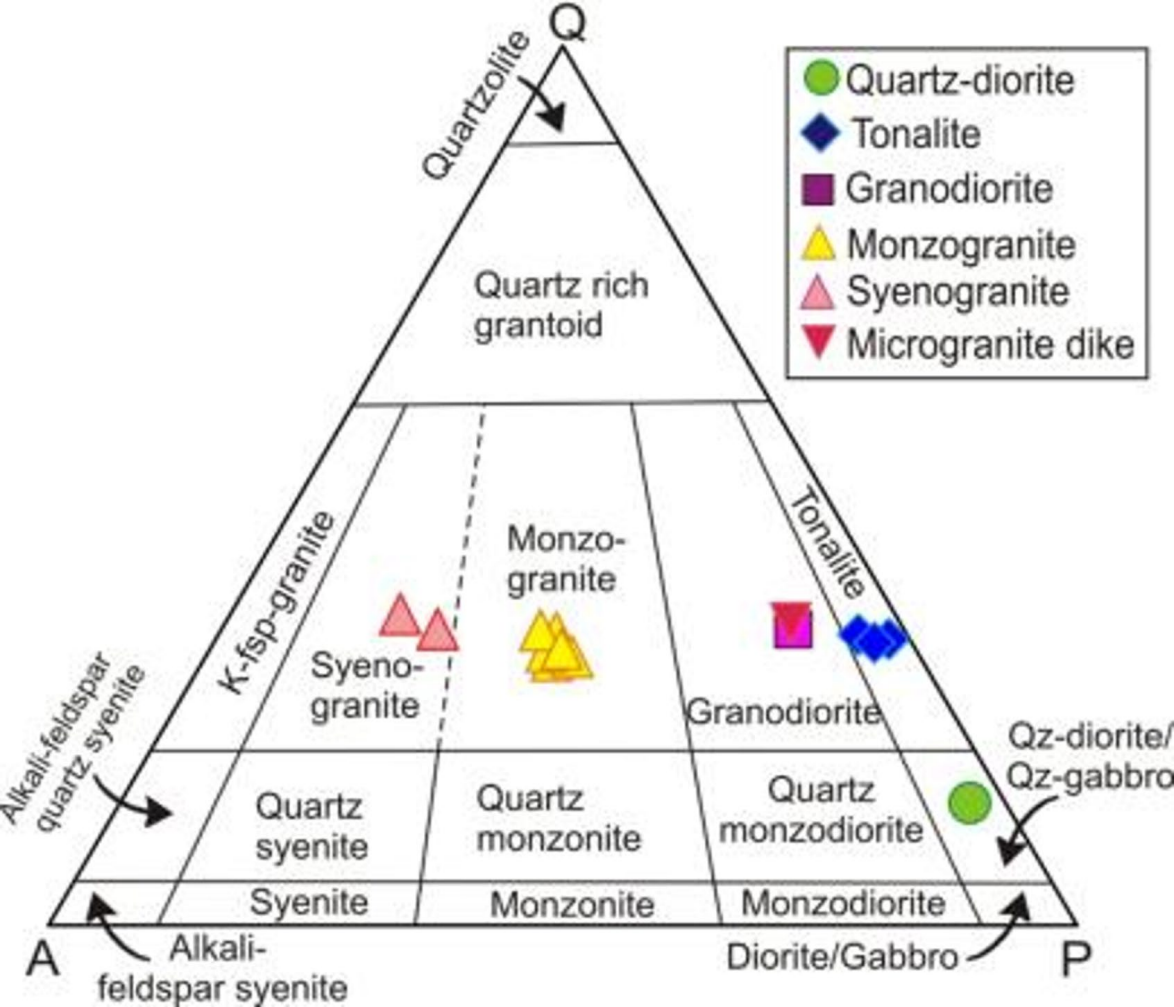
Modal QAP classification of Abu Murrat granitoids, after Streckeisen^32^. Syn-orogenic granites plot as quartz diorites, tonalites, and granodiorites. Post-orogenic granites plot as monzogranites, with minor syenogranite.

**Fig. 4:**
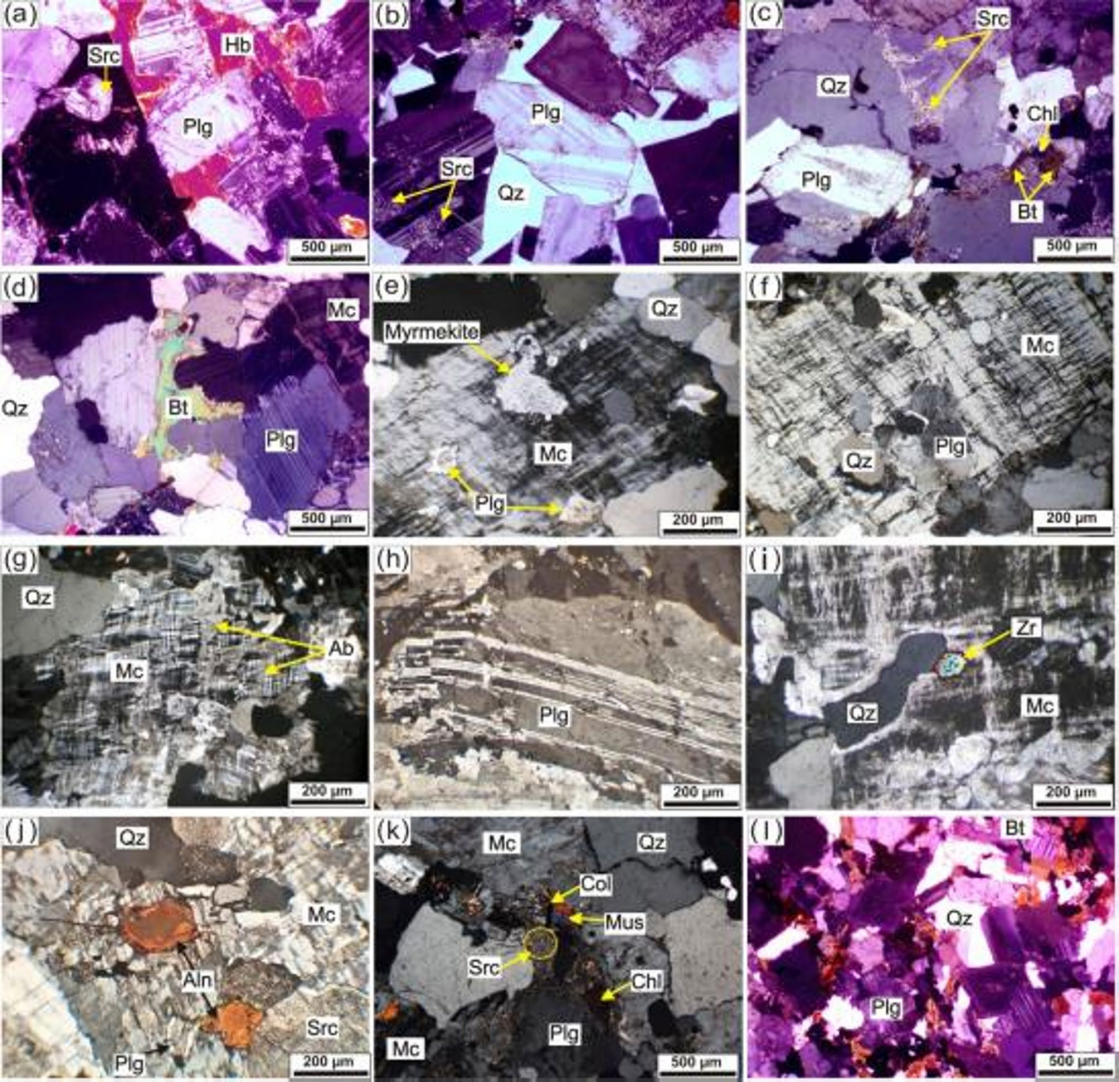
Photomicrographs of Abu Murrat granitoids. (**a**) Quartz diorite: sericitized plagioclase (Plg) crystals pseudo-ophitically enclosed in twinned hornblende (Hb) oikocryst. (**b**) Quartz diorite: interstitial quartz (Qz) filling space between zoned, sericitized plagioclase (Plg) crystals. (**c**) Tonalite: cracked quartz (Qz) and biotite (Bt) flakes between partly sericitized plagioclase (Plg) in a hypidiomorphic texture. (**d**) Granodiorite: interstitial biotite (Bt) between plagioclase (Plg), microcline (Mc), and quartz (Qz). (**e**) Monzogranite: myrmekitic intergrowth (vermicular quartz in feldspar), quartz, and plagioclase hosted within large microcline crystal. (**f**) Monzogranite: large poikilitic microcline (Mc) enclosing corroded plagioclase (Plg) and quartz (Qz). (**g**) Monzogranite: anhedral microcline (Mc) grain hosting irregular albite lamellae (Ab). (**h**) Monzogranite: large plagioclase (Plg) showing deformed (offset) albite twins. (**i**) Monzogranite: polygonal zircon (Zr) and quartz (Qz) hosted in microcline (Mc). (**j**) Monzogranite: prismatic allanite (Aln) crystal (deep red-brown) hosted in plagioclase (Plg). (**k**) Monzogranite: fine columbite (Col) inclusion within microcline (Mc). (**l**) Microgranite dike: twinned and zoned plagioclase with interstitial quartz (Qz) and biotite (Bt).

The syn-to-late orogenic granite suite in Abu Murrat includes quartz diorite, tonalite, and granodiorite, classified by modal mineral proportions (Supplementary Table 1; Fig. 3), which were determined through petrographic point counting of representative thin sections. Quartz diorite is medium- to coarse-grained with a hypediomorphic granular texture. Its essential mineralogy is dominated by plagioclase (often exhibiting pericline twinning and local zoning; 55‒60 vol%) and hornblende (15‒20 vol%), with subordinate quartz (10‒15 vol%) and biotite (~ 5 vol%) (Fig. 4a, b). Accessory phases observed in the syn-orogenic granitoids include Fe–Ti oxide minerals, sphene, zircon, and apatite; however, not all of these phases are visible in the representative photomicrographs shown in Fig. 4a, b. Secondary chlorite and sericite occur from alteration of biotite and plagioclase (Fig. 4a, b). Tonalite is texturally similar but has higher quartz (25‒30 vol%) and biotite (~ 10 vol%) and slightly lower plagioclase (50‒55 vol%) and hornblende (5‒10 vol%) compared to quartz diorite, with minor interstitial K-feldspar (orthoclase or microcline) (Fig. 4c). Granodiorite shows a further increase in quartz (25‒30 vol%) and K-feldspar (including microperthite; 15‒20 vol%), with plagioclase (40‒45 vol%) and mafic minerals (biotite ± hornblende; 5‒10 vol%) (Fig. 4d). Granodioritic samples commonly contain accessory muscovite and allanite in addition to zircon, apatite, and iron oxides. Plagioclase is occasionally partially sericitized (especially in grain cores), hornblende is locally replaced by biotite ± chlorite, and quartz shows undulatory extinction and microcracks, indicating mild strain (Fig. 4a‒d). Although minor sericite and chlorite alterations are observed, the alteration is weak and is not considered to have significantly affected the primary mineralogical or geochemical characteristics of the rocks.

The post-orogenic granite suite is largely monzogranite (with subordinate syenogranite) and is typically coarse-grained, massive, and exhibits an allotriomorphic (inequigranular) texture (Fig. 4e–k). The monzogranite consists of abundant alkali feldspar (orthoclase microperthite and microcline; 25‒30 vol%) and plagioclase (often as large, subhedral crystals; 30‒35 vol%), with interstitial quartz (30‒35 vol%) and ~ 5% ferromagnesian minerals mainly as biotite (Fig. 4e–k). Feldspars commonly show graphic to myrmekitic intergrowths with quartz (Fig. 4e, f, i), and K-feldspar megacrysts frequently enclose lath-shaped plagioclase, rounded quartz, and biotite (Fig. 4e, f, i). Microcline occasionally hosts albite lamellae forming mesoperthite and perthite textures (Fig. 4g). Plagioclase also occurs as large plate-like grains commonly show albite twinning, which is locally deformed in some crystals (Fig. 4h). Biotite occurs as anhedral flakes, often partially altered along cleavage to chlorite, and is typically accompanied by secondary muscovite. The fine-grained white mica is interpreted as secondary sericite/muscovite, developed mainly through feldspar replacement during late- to post-magmatic hydrothermal sericitization. No petrographic evidence for primary magmatic muscovite was identified in the studied suite. Accessory minerals in the monzogranite include zircon, commonly occurring as small prismatic to polygonal grains with pleochroic halos in biotite (Fig. 4i), and allanite, observed as short reddish-brown, locally zoned prisms (Fig. 4j). Titanite, monazite, and possible Nb–Ta oxides of the columbite–tantalite series are also present or suspected (Fig. 4k), although the latter phases require microanalytical confirmation. The syenogranite is petrographically similar but contains a higher modal proportion of K-feldspar (40–45%) and correspondingly less plagioclase (~ 10–15%), consistent with its more alkaline composition.

The late microgranite dikes are medium-grained and occasionally porphyritic. They are composed chiefly of plagioclase (~ 55–60 vol%), quartz (~ 20–25 vol%), and biotite (~ 15–20 vol%), with minor potassic feldspar (mostly microcline-perthite) typically < 10 vol% (Fig. 4l). Plagioclase occurs both as short groundmass laths and as longer prismatic phenocrysts, generally showing normal zoning, sometimes with sericitized cores and fresh rims (Fig. 4l). Anhedral quartz and tiny flakes of biotite occupy the interstitial spaces, and biotite is occasionally partly chloritized. These microgranite dikes share mineralogical characteristics with the post-orogenic granite suite, though their emplacement along shear zones and stronger alteration suggest significant late-stage hydrothermal interaction^26^.

## Methods

Mineral compositions were determined using a JEOL JXA-8230 electron probe microanalyzer at the Earth Sciences Application and Research Center (YEBİM), Ankara University (Supplementary Table 2). Electron probe microanalysis (EMPA) data were obtained from 15 polished thin sections representing the main lithologies of the Abu Murrat granitoid suites. A total of 148 point analyses were collected from feldspar, mica, chlorite, Fe–Ti oxide, zircon and Nb–Ta oxide minerals. Analytical spots were selected after transmitted- and reflected-light petrographic examination. Analyses were performed at 20 kV accelerating voltage and 20 nA beam current, with a focused beam (1–5 μm) and counting times of 10–60 s per element. The instrument was calibrated using a suite of natural and synthetic standards (minerals and oxides) spanning the elements Na through Zr. A Phi–Rho–Z matrix correction (CITZAF)^33^ was applied to raw data. Analytical precision is better than ~ 1–2% (relative) for concentrations > 1 wt%, and ~ 5–10% for those below that threshold. Mineral formulae were recalculated using mineral-specific normalization schemes: feldspars (8 O); micas (22 O); chlorite (28 O with stoichiometric H_2_O); zircon (4 O); columbite–tantalite minerals (6 O); magnetite (4 O); hematite (3 O); and goethite (3 O; O + OH,, assuming FeO(OH)). The normalization basis is detailed in Supplementary Table 2 for each mineral group.

Fourteen representative, fresh and least-altered samples from the syn-to-late orogenic and post-orogenic granite suites were selected for whole-rock geochemical analysis at the Geochemistry Research Laboratories of Istanbul Technical University (ITU/JAL) (Supplementary Table 3). Prior to analysis, samples were crushed, dried, and homogenized to obtain representative powders. Following the ITU/JAL preparation protocol, dried sample aliquots were powdered using a tungsten-carbide milling assembly to achieve a homogeneous grain size suitable for whole-rock XRF and ICP-MS analysis. Possible contamination related to this preparation route was specifically considered because tungsten-carbide milling may introduce W, Co and, to a lesser extent, minor transition-metal contributions. Analytical quality control was evaluated using the USGS GSP-2 granodiorite certified reference material, which was prepared using the same tungsten-carbide milling procedure as the unknown samples and analyzed in the same analytical workflow. Accordingly, possible W, Co, and minor transition metals contamination from sample preparation is acknowledged but it is not considered to significantly affect the major oxides, REE patterns, or immobile-element parameters used for interpretations. W and Co were not used as petrogenetic indicators, and Ta-based discrimination parameters were treated cautiously. Major oxides were measured by wavelength-dispersive X-ray fluorescence (XRF) using a Bruker S8 TIGER spectrometer. Trace elements and rare earth elements (REE) were determined by inductively coupled plasma mass spectrometry (ICP-MS) on a PerkinElmer ELAN DRC-e instrument. For ICP-MS analysis, powdered samples were digested following the ITU/JAL mixed-acid digestion procedure using HCl, HNO₃, HF and H₃BO₃ prior to instrumental measurement. Analytical precision is expressed as relative standard deviation (RSD) based on replicate measurements, whereas accuracy was evaluated from the deviation of measured CRM values from certified or recommended concentrations. Under typical operating conditions, major oxides yielded ≤ ~ 5% RSD, although uncertainties may be higher near detection limits and for volatile components. Most trace elements and REE yielded ≤ ~ 10% RSD, with higher uncertainties expected for concentrations close to method detection limits or for elements affected by spectral or polyatomic interferences. Eu anomalies were calculated as Eu/Eu*= Eu_N_/(Sm_N_ x Gd_N_)^0.534^. The La_N_/Lu_N_ and La_N_/Yb_N_ ratios were normalized to chondritic compositions using the normalization constants of McDonough and Sun^35^.

## Results

### Mineral chemistry

**Fig. 5:**
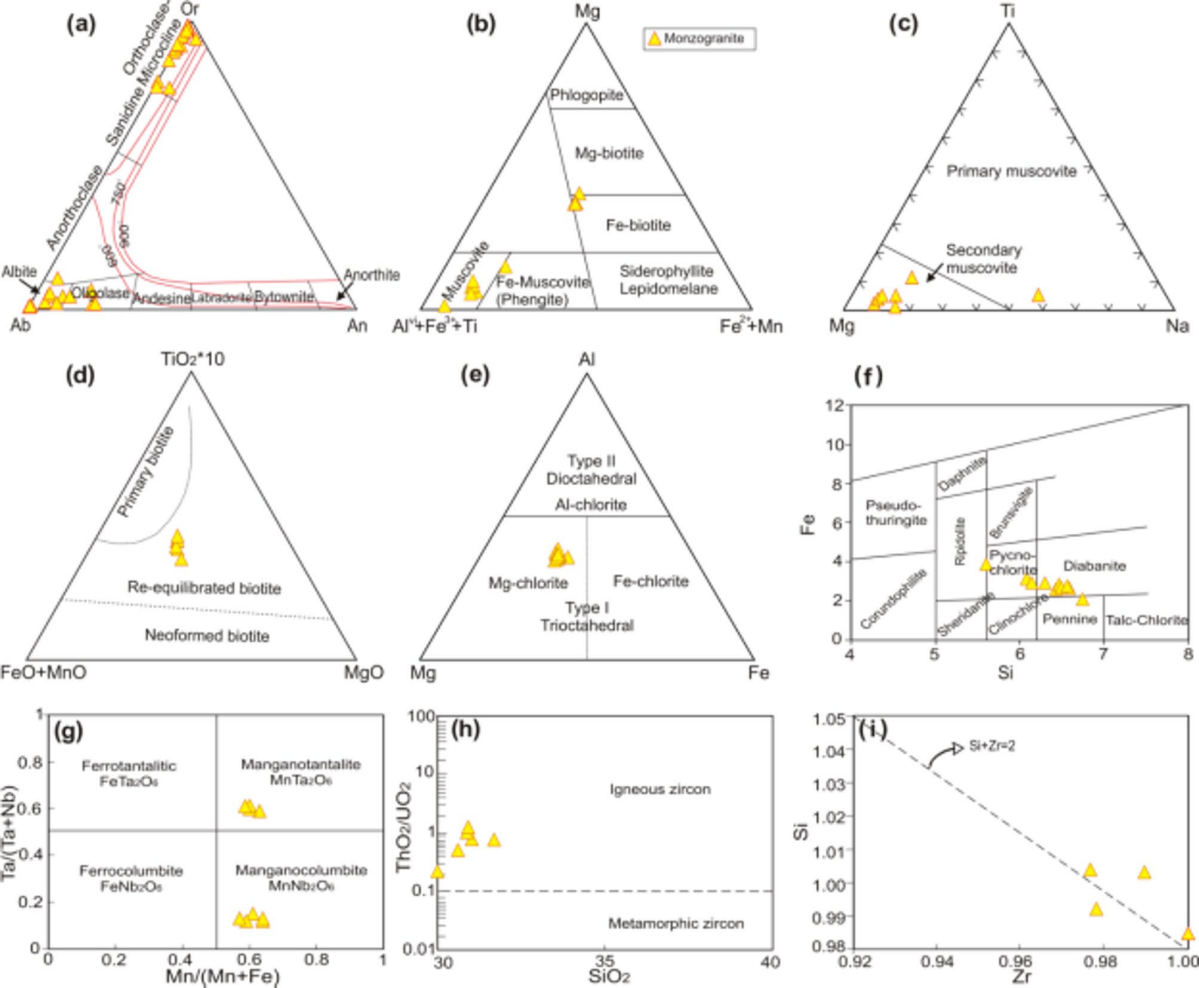
Mineral chemistry of Abu Murrat granitoids. (**a**) Feldspar compositions in Ab–Or–An ternary diagram^43^. (**b**) Mica classification after Foster^36^. (**c**) Mg–Ti–Na ternary distinguishing primary vs. secondary muscovite^37^. (**d**) Biotite composition in TiO_2_ × 10–(FeO^t^+MnO)–MgO ternary diagram^44^ showing re-equilibrated vs. primary fields. (**e**) Al–Mg–Fe diagram for chlorite classification^38^. (**f**) Si vs. Fe diagram^45^ showing diabantite with minor pycnochlorite composition of chlorite. (**g**) Ta/(Nb + Ta) vs. Mn/(Mn + Fe) for Nb–Ta oxides^46^, indicating manganotantalite and manganocolumbite. (**h**) ThO_2_/UO_2_ vs. SiO_2_ in zircon^41^. (**i**) Zr site cations vs. Si site cations in zircon; dashed line indicates ideal Zr + Si=2 stoichiometry^42^.

The feldspars in the post-orogenic granite (monzogranite) are compositionally homogeneous alkali feldspar and plagioclase. Microprobe analyses show K-feldspar with 15.38–19.03 wt% Al_2_O_3_ and 12.69–14.62 wt% K_2_O, corresponding to an orthoclase component (Or_75.8–97.6_) and very low anorthite (An_0–5.2_) and albite (Ab_2.4–22.5_) content (Supplementary Table 2, Fig. 5a). Plagioclase in the monzogranite is albite-rich (Ab_77.9–99.8_) with high SiO_2_ (61.13–77.23 wt%) and Al_2_O_3_ (18.8–24.27 wt%) contents (Supplementary Table 2, Fig. 5a). Alteration of feldspar to fine sericite is common; the sericite has high K_2_O (7.2–8.8 wt%) and low Na_2_O (< 0.2 wt%) (Supplementary Table 2).

Micas in the monzogranite include primary magmatic biotite and secondary muscovite. Secondary muscovite is compositionally uniform, with mean SiO_2_ = 44.45 wt%, Al_2_O_3_ = 29.90 wt%, FeO = 4.49 wt%, MgO = 1.73 wt%, and K_2_O = 10.81 wt% (Supplementary Table 2). In the Mg–(Fe^2+^+Mn)–(Fe^3+^+Al^IV^+Ti) ternary diagram^36^, most analyses fall in the muscovite field, with one plotting in the Fe-rich muscovite (phengite) field (Fig. 5b). On the Mg–Ti–Na diagram^37^, compositions match secondary muscovite and are supported by petrographic textures (Fig. 5c). The biotite in the monzogranite is Fe-rich (ferroan), with average ~ 38.4 wt% SiO2, 14.6 wt% Al_2_O_3_, 16.6 wt% FeO, 12.5 wt% MgO, 1.9 wt% TiO_2_, and 10.4 wt% K_2_O (Supplementary Table 2). In classification, it falls in the re-equilibrated (late-magmatic) biotite field (Fig. 5d), suggesting that it experienced some subsolidus or hydrothermal alteration, as evidenced by partial chloritization of biotite. Chlorite replacing biotite in the monzogranite is classified as Mg-rich chlorite, plotting in the Mg–Al–Fe trioctahedral field (Fig. 5e)^38^. Moreover, it is classified as diabantite with minor pycnochlorite, as illustrated by Si versus Fe_2+_ + Fe_3+_ diagram of Hey (1954) (Fig. 5f).

Accessory minerals provide additional petrogenetic information. Nb–Ta oxide minerals in the monzogranites belong to the columbite–tantalite group and define two compositional populations on the Ta/(Nb + Ta) versus Mn/(Mn + Fe) quadrilateral (Fig. 5g; Supplementary Table 2). The first population consists of Nb-rich manganocolumbite analyses (*n* = 5), with Ta/(Ta + Nb) = 0.12–0.15 and Mn/(Mn + Fe) = 0.57–0.64. These analyses have Nb_2_O_5_ = 61.32–64.66 wt% (mean ≈ 62.85 wt%) and Ta_2_O_5_ = 14.45–17.44 wt% (mean ≈ 15.50 wt%), MnO = 11.27–12.61 wt% (mean ≈ 11.80 wt%) and FeO = 6.92–8.45 wt% (mean ≈ 7.66 wt%) (Supplementary Table 2; Fig. 5g). The second population consists of Ta-rich manganotantalite analyses (*n* = 4), with Ta/(Ta + Nb) = 0.59–0.61 and Mn/(Mn + Fe) = 0.59–0.63 (mean ≈ 0.61) (Supplementary Table 2; Fig. 5g). These analyses have Nb_2_O_5_ = 21.93–23.64 wt%, Ta_2_O_5_ = 57.44– 58.23 wt%, MnO = 10.16–10.67 wt% and FeO = 6.41–7.22 wt%. The coexistence of Nb-rich columbite and Ta-rich tantalite is consistent with progressive Nb–Ta fractionation during late-stage melt evolution, locally modified by fluid-assisted replacement^39,40^. Zircon occurs as both primary magmatic grains and altered, porous grains in the monzogranite. Fresh magmatic zircons are high in ZrO_2_ (53–64 wt%) and SiO_2_ (~ 30 wt%), with low P_2_O_5_ (~ 0.1 wt%) (Supplementary Table 2). In contrast, altered zircon shows elevated P_2_O_5_ (1.3–2.7 wt%) and UO_2_ (~ 1.0 wt%) with lower ZrO_2_ (36–48 wt%) and SiO_2_ (22–28 wt%) (Supplementary Table 2), indicating interaction with late-stage magmatic fluids. On discrimination diagrams, the P-free zircons plot in the igneous zircon field^41^ and lie near the ideal Zr: Si stoichiometric line^42^ (Fig. 5h–i), confirming their magmatic origin, whereas the P-bearing altered zircons deviate due to xenotime-type substitution. Fe–Ti oxides in the monzogranite are dominantly magnetite (often titaniferous, up to ~ 2.3 wt% TiO_2_) (Supplementary Table 2) with minor secondary hematite/goethite along grain margins. Magnetite compositions vary from nearly pure Fe_3_O_4_ (FeO ~ 86–99 wt%) to Ti-bearing magnetite (Supplementary Table 2). Secondary hematite and goethite have lower FeO (~ 74–77 wt% and 51–56 wt%, respectively) (Supplementary Table 2), reflecting oxidation during late-stage alteration.

### Whole-rock geochemistry

Major element analyses (Supplementary Table 3) reveal clear distinctions between the syn-to-late and post-orogenic granite suites. The post-orogenic granites (monzogranites) have higher SiO_2_ (70.2–75.8 wt%) and K_2_O (5.1–8.0 wt%), and lower Fe_2_O_3_ (0.9–1.9 wt%), MgO (0.04–0.62 wt%), CaO (0.6–1.5 wt%), and Na_2_O (3.0–4.5 wt%) compared to the syn-to-late orogenic granites (Supplementary Table 3). Syn-to-late orogenic granite samples span SiO_2_ = 59.7–69.3 wt% with K_2_O = 0.9–2.3 wt%, Fe_2_O_3_ = 1.5–4.8 wt%, MgO = 0.6–2.2 wt%, CaO = 4.0–7.3 wt%, and Na_2_O = 4.7–5.7 wt% (Supplementary Table 3). An outlier leucogranite sample is extremely enriched in SiO_2_ (~ 89.8 wt%) and accordingly depleted in other oxides (Supplementary Table 3). Because of its extremely high SiO_2_ content, this quartz-rich leucogranite is treated separately from the main monzogranite population. Its composition may reflect quartz enrichment, silicification or local quartz veining in addition to magmatic differentiation. The sample is retained in the diagrams for transparency, but it is not used to define the principal fractionation trends of the post-orogenic suite. Conversely, a microgranite dike sample has the lowest SiO_2_ (~ 56.1 wt%) and the highest TiO_2_, Fe_2_O_3_, and P_2_O_5_ of all samples (Supplementary Table 3), reflecting its less evolved, more mafic character.

**Fig. 6:**
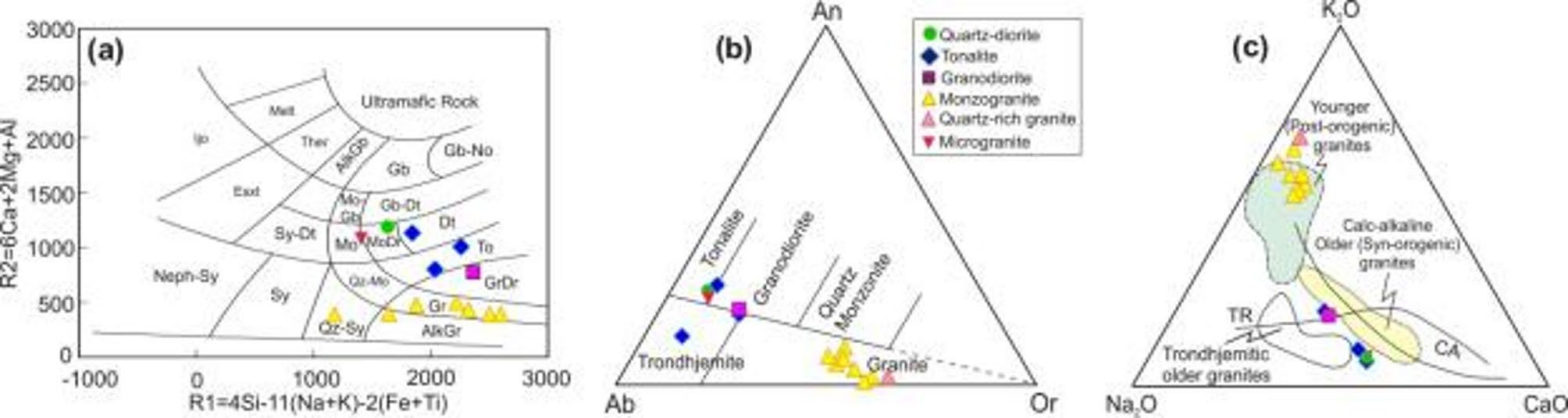
(**a**) R1–R2 chemical classification diagram^47^ for Abu Murrat granitoids. (**b**) Normative An–Ab–Or ternary^48^. (**c**) K_2_O–Na_2_O–CaO ternary showing fields of Egyptian granitoids, after Hassan and Hashad^49^. Trondhjemitic (TR) and calc-alkaline (CA) trends from Barker and Arth^50^. AlkGr: Alkali Granite, Gr: Granite, GrDr: Granodiorite, To: Tonalite, Dt: Diorite, MoDr: Monzodiorite, Gb: Gabbro, Gb-Dt: Gabbro-diorite, Gb-No: Gabbro-Norite, AlkGb: Alkali Gabbro, Mo-Gb: Monzo-Gabbro, Sy-Gb: Syeno-Gabbro, Sy-Dt: Syeno-Diorite, Mo: Monzonite, Qz-Mo: Quartz-Monzonite, Sy: Syenite, Qz-Sy: Quartz-Syenite, Neph-Sy: Nepheline Syenite, Esxt: Essexite, Ijo: ijolite, Ther: theralite, Melt: Melteigite.

**Fig. 7:**
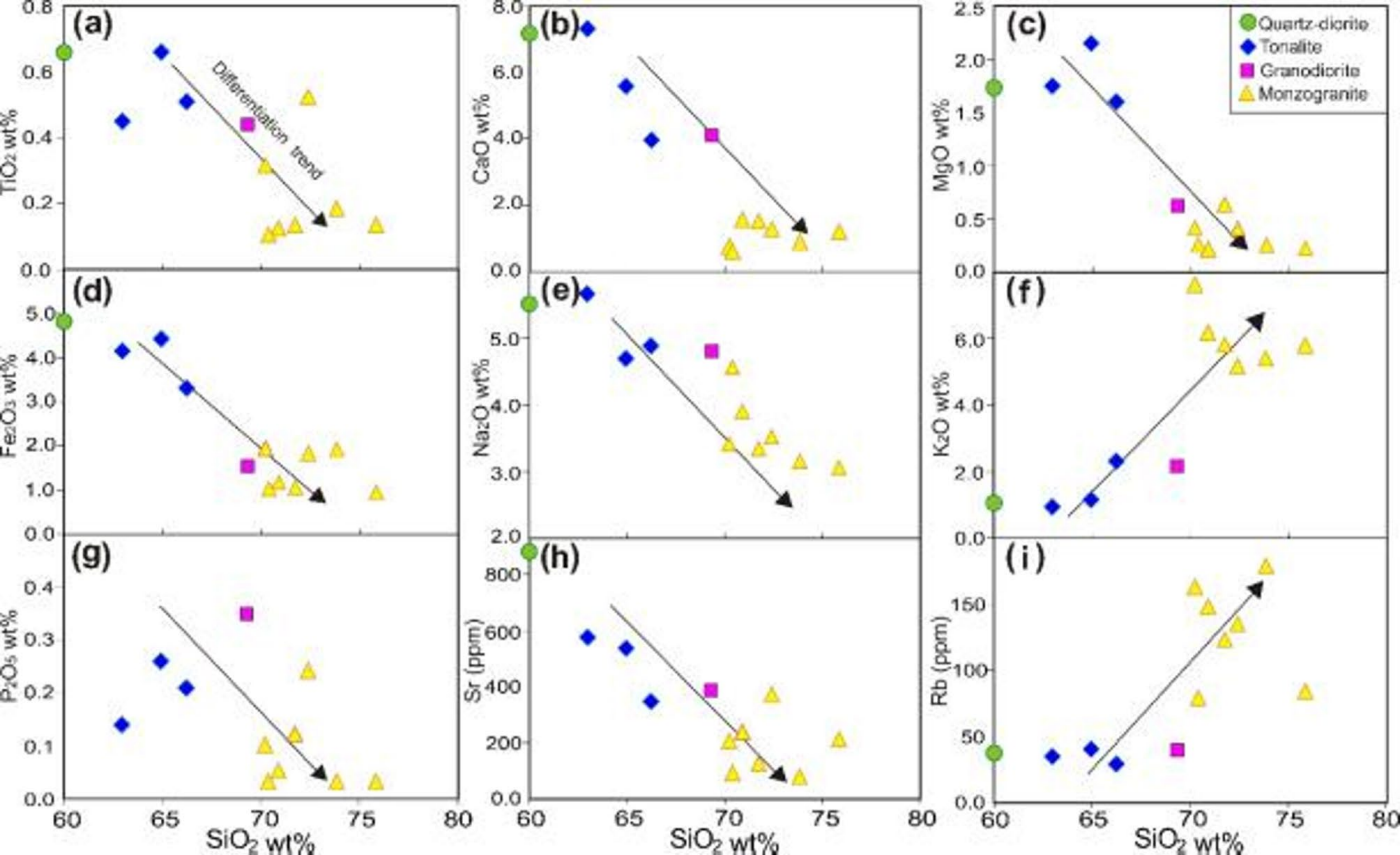
Harker variation diagrams of selected major oxides and trace elements vs. SiO_2_ for Abu Murrat granitoids.

Chemical classification diagrams underscore these differences. In the R1–R2 multicationic plot of De la Roche, et al.^47^, syn-orogenic granite compositions fall in the tonalite, diorite, and monzodiorite fields (Fig. 6a), whereas the post-orogenic granites plot predominantly in the granite field (with two samples in alkali granite or quartz syenite fields) (Fig. 6a). Normative Ab–An–Or ternary plot^48^ similarly place the syn-orogenic granite samples within granodiorite–tonalite fields, and the post-orogenic granite samples firmly in the granite field (Fig. 6b). Based on the major oxides Na_2_O, K_2_O, and CaO contents^49^, the monzogranites and quartz-rich leucogranite show affinity with Egyptian Younger Granites, whereas the quartz diorite–tonalite–granodiorite samples correspond to Egyptian Older Granite compositions (Fig. 6c). Harker variation diagrams (Fig. 7) illustrate the control of fractional crystallization. With increasing SiO_2_, TiO_2_, FeOₜ, MgO, CaO, Na_2_O, P2O_5_, and Sr generally decrease, whereas K_2_O and Rb tend to increase (Fig. 7). These variations are interpreted as qualitative first-order differentiation trends because the dataset includes different lithological groups and a quartz-rich outlier. Despite minor post-magmatic alteration observed petrographically, there is no evidence of significant element mobility, and the major-element geochemical trends are considered to reflect primary magmatic processes.

**Fig. 8:**
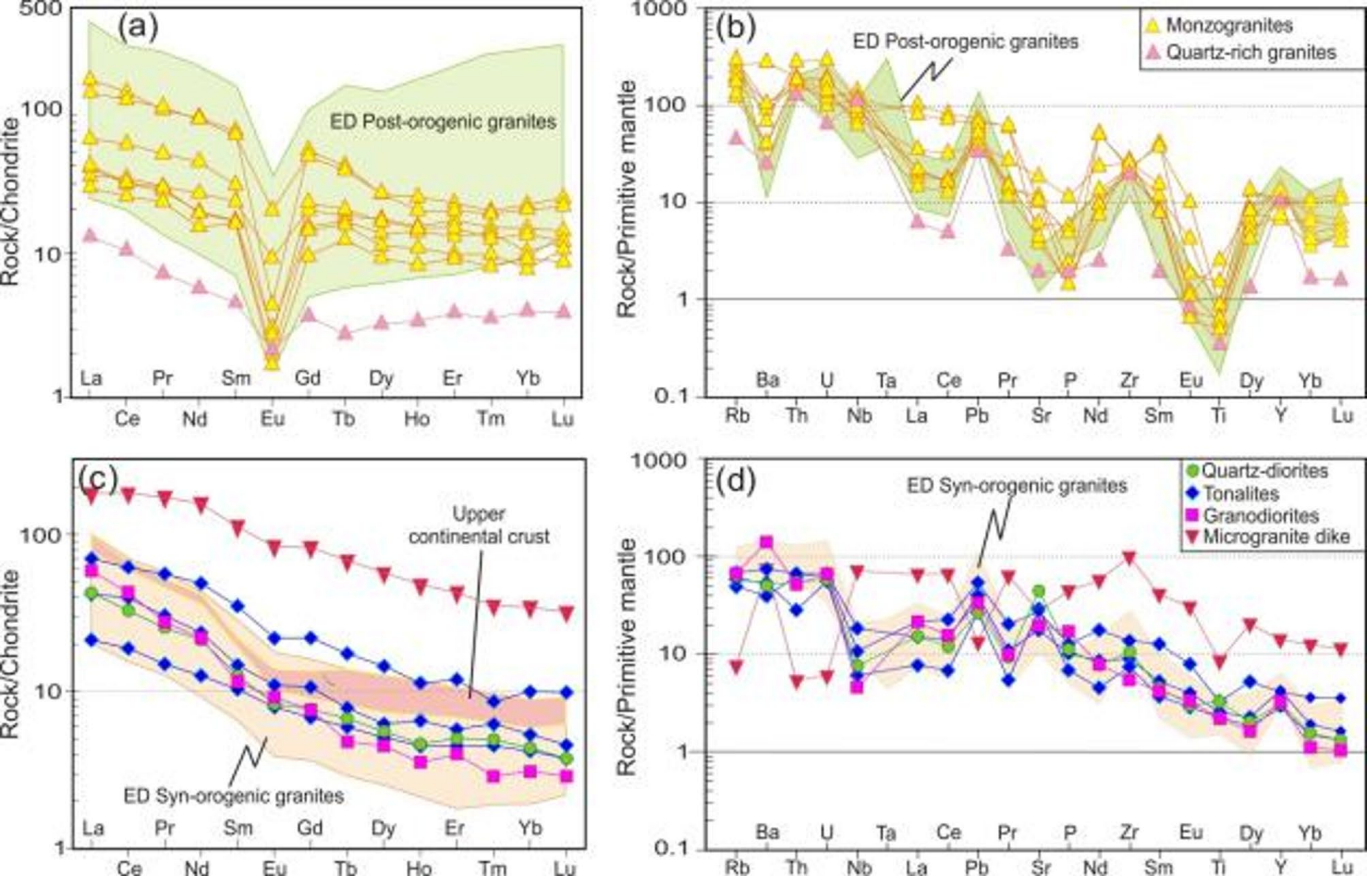
Whole-rock geochemistry of Abu Murrat granites. (**a**, **c**) Chondrite-normalized REE patterns of syn-orogenic vs. post-orogenic granites. (**b**, **d**) Primitive mantle-normalized multi-element diagrams of syn-orogenic vs. post-orogenic granites. Normalization values from McDonough and Sun^35^. Fields for Eastern Desert post-orogenic (A-type) and syn-orogenic (I-type) granites from Azer, et al. ^5^, El-Awady, et al. ^18^, Sami, et al.^57^.

Trace element patterns further distinguish the suites. The monzogranites have significantly higher total rare earth element (REE) contents (ΣREE up to ~ 342 ppm) than the syn- to late orogenic granite varieties (ΣREE ~ 98 ppm) (Supplementary Table 3). Chondrite-normalized REE plots (Fig. 8a, c) for the monzogranites show steep light REE enrichment and a deep negative Eu anomaly (Eu/Eu = 0.05–0.39) (Supplementary Table 3), typical of highly fractionated A-type granites. These REE patterns closely resemble those of other post-tectonic A-type granites in the Eastern Desert of Egypt (e.g., Homrit Waggat, Gabal El-Ineigi, Gabal Abu Kibash) (Fig. 8a, c)^18,51,52^. The quartz-rich granite is treated separately because it’s very high SiO_2_ content, low total REE abundances, and subdued Eu anomaly distinguish it from other samples. These features may reflect quartz enrichment, local silicification, or derivation from a highly evolved leucocratic melt. However, these interpretations remain tentative because accessory-mineral behavior, melt extraction, and Si-metasomatism can significantly modify REE systematics in felsic rocks^53–56^. The syn-orogenic granites show higher overall LREE/HREE ratios (La_N_/Lu_N_ = 5.7–20.4) but only a small negative Eu anomaly (Eu/Eu ~ 0.8–1.0) (Supplementary Table 3), consistent with minimal plagioclase removal under water-rich conditions. Primitive mantle-normalized multi-element diagrams (Fig. 8b, d) highlight that the syn-orogenic granites are enriched in LILE (Rb, Ba, Sr) relative to HFSE (Nb, Zr, Ti), matching the signature of arc-related I-type granites. In contrast, the post-orogenic granites display pronounced negative anomalies in Ba, Sr, P, and Ti and positive anomalies in Rb, Th, U, Pb, Zr, and Y (Fig. 8a, c), which are characteristic of intraplate A-type granites.

## Discussion

### Magmatic crystallization temperatures

Accessory minerals (zircon, monazite, apatite) host key trace elements (Zr, LREE, P) whose solubility in melt is highly temperature-dependent. Assuming the magmas were saturated in these phases, whole-rock Zr, LREE, and P concentrations can be used to estimate crystallization temperatures^58–60^. The syn- and post-orogenic granite suites of Abu Murrat reflect fundamentally different magmatic conditions. Zirconium contents are higher in the post-orogenic granites (A-type monzogranites, avg. ~246 ppm) than in the syn-orogenic granites (I-type, avg. ~96 ppm), implying higher melt temperatures for the former. Indeed, zircon saturation thermometry^60^ yields ~ 794–835 °C for the post-orogenic suite versus ~ 635–731 °C for the syn-orogenic suite (Supplementary Table 3). These values are consistent with independent estimates for Eastern Desert A-type and I-type granites^10,25,61^ and suggest the A-type magmas were generated by high-temperature crustal melting capable of dissolving even refractory zircon^62^.

**Fig. 9:**
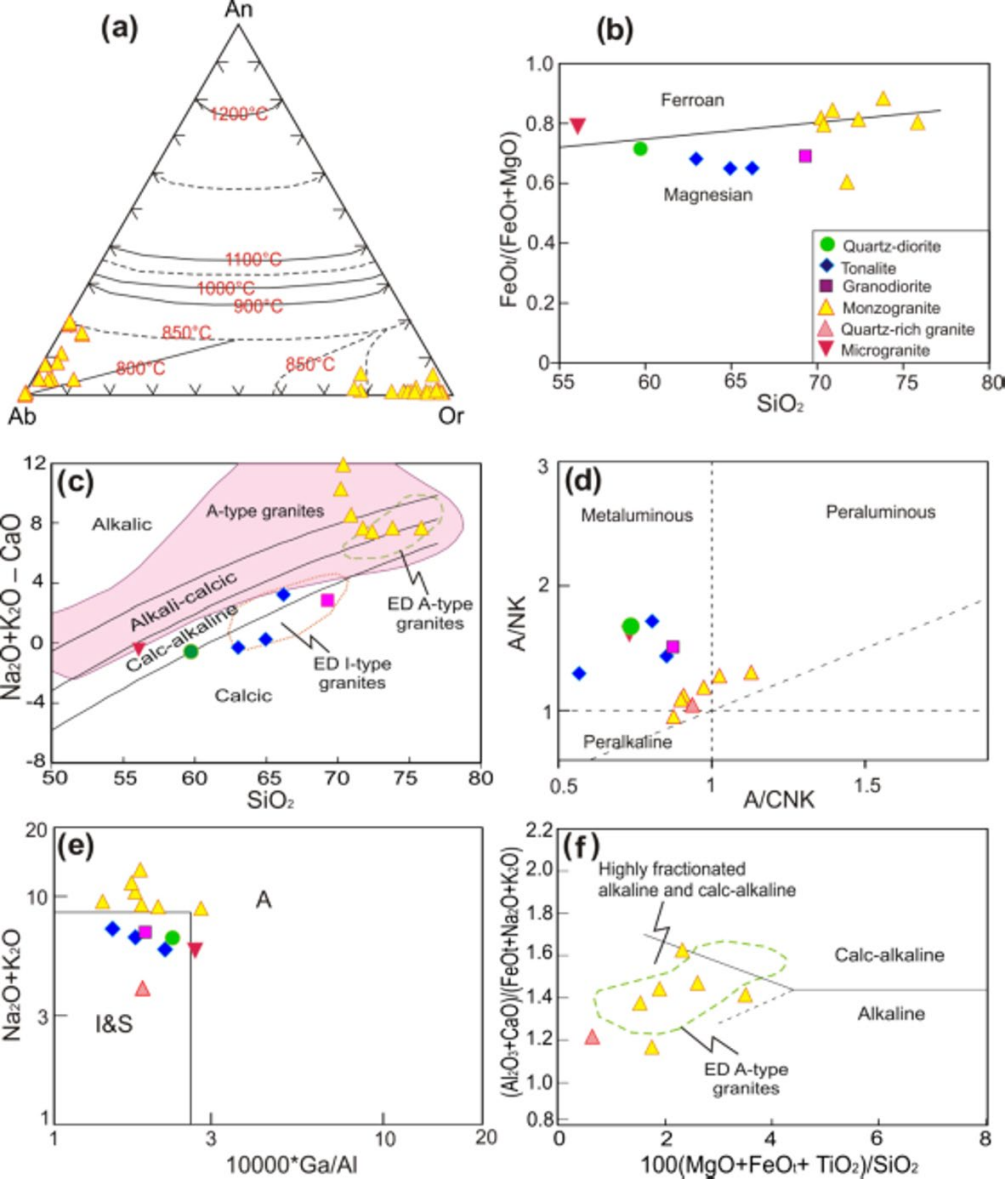
Geochemical discrimination diagrams. (**a**) Ab–An–Or ternary showing feldspar thermometry (~ 800–850 °C) ^65^. (**b**) FeO^t^/(FeO^t^+MgO) vs. SiO_2_, showing syn-orogenic granites as magnesian and post-orogenic granites as ferroan^67^. (**c**) (Na_2_O + K_2_O) – CaO vs. SiO_2_ illustrating calc-alkaline vs. alkali-calcic trends^67^. (**d**) A/NK vs. A/CNK (alumina saturation)^68^ discriminating metaluminous (syn-orogenic) vs. peraluminous (post-orogenic) nature. (**e**) 10,000×Ga/Al vs. Na_2_O+K_2_O^69^, showing syn-orogenic granites in I- & S-type field and post-orogenic granites in A-type field. (**f**) (Al_2_O_3_+CaO)/(FeO+Na_2_O+K_2_O) vs. 100×(MgO+FeO^t^+TiO_2_)/SiO_2_^70^, with post-orogenic monzogranites plotting in highly fractionated alkaline & calc-alkaline field similar to Eastern Desert Younger Granites.

Monazite saturation temperatures^59^ were calculated assuming a melt H_2_O content of ~ 3 wt%. This value is considered moderate for hydrous granitoid melts and is consistent with the presence of biotite ± hornblende in the syn-orogenic rocks, as well as geochemical evidence for feldspar fractionation. Because no direct melt H_2_O measurements are available, the monazite temperatures should be regarded as model-dependent estimates rather than absolute crystallization temperatures^63,64^. These monazite-based temperatures are also higher for the post-orogenic granites (632–862 °C; avg. ~727 °C) than for the syn-orogenic granites (502–666 °C; avg. ~615 °C) (Supplementary Table 3). They are systematically lower than the corresponding zircon saturation values, indicating that zircon crystallized at higher temperatures (earlier in the cooling history) and confirming the reliability of both thermometers. The two-feldspar thermometry^65^ from coexisting plagioclase–K-feldspar pairs further indicates crystallization around 800–850 °C (Fig. 9a), consistent with the high-temperature regime of these granitoids. Finally, subsolidus re-equilibration temperatures of biotite were estimated using the equation of Henry, et al.^66^, yielding values ranging from 587 to 638 °C. These temperatures likely reflect post-magmatic re-equilibration rather than primary magmatic crystallization condition.

### Genetic classification of the granitoids

**Fig. 10:**
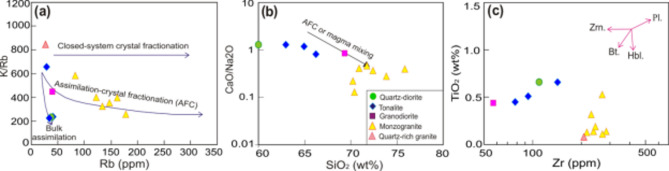
Geochemical evidence for contamination and fractional crystallization. (**a**) K/Rb vs. Rb plot^75^ showing assimilation (low Rb, moderate K/Rb) trend for syn-orogenic granites vs. fractional crystallization (high Rb, high K/Rb) trend for post-orogenic granites. (**b**) CaO/Na_2_O vs. SiO_2_^75^ showing distinct AFC trajectories for syn-orogenic vs. post-orogenic suites. (**c**) Zr vs. TiO₂ plot indicating plagioclase-dominated fractionation path. These plots illustrate qualitative trends and are not quantitative AFC models.

The geochemical features of the Abu Murrat granitoids confirm that the syn-orogenic granites are typical subduction-related I-type granites, whereas the post-orogenic granites display characteristics of intraplate A-type granites. The syn-orogenic granite suite contains hornblende and biotite, is largely metaluminous (A/NK > 1 and A/CNK < 1) and lacks significant normative corundum (Supplementary Table 3). These features are consistent with an I-type, mantle-influenced lineage that experienced some crustal assimilation (Fig. 9b–e). Its relatively low SiO_2_ and high CaO, MgO, and Al_2_O_3_ contents, along with enrichment in LILE over HFSE, point to genesis in an arc environment from hydrous basaltic magmas that partially melted and assimilated continental crust (Figs. 8d and 10). In contrast, the post-orogenic monzogranites are rich in silica and alkalis, marginally peraluminous (some samples with A/CNK > 1) (Supplementary Table 3; Fig. 9d) and contain iron-rich biotite (Fig. 5b) but no amphibole—hallmarks of A-type (anorogenic) granites. High (FeO^t^/(FeO^t^+MgO) ≈ 0.8) values place them in the ferroan granite field (Fig. 9b), and they plot as alkali-calcic to alkalic in composition (Fig. 9c), unlike the calc-alkaline syn-orogenic granites (Fig. 9c). High Ga/Al ratios and elevated HFSE concentrations (Nb, Zr, Y, Th) (Supplementary Table 3) further support their A-type affinity (Fig. 9e). The distinct negative Eu anomalies in the post-orogenic granites (Fig. 8a) reflect extensive fractional crystallization of plagioclase (and likely K-feldspar) from an initially H_2_O-poor melt. Meanwhile, the very weak Eu anomalies in the syn-orogenic granites (Fig. 8c) imply that plagioclase remained in the melt until late, consistent with higher H_2_O buffering suppressing Eu depletion.

### Magmatic differentiation and crustal assimilation

The crustal assimilation and fractional crystallization (AFC) model is inferred from integrated major- and trace-element behavior systematic rather than from any single discrimination plot. In particular, fractional crystallization is indicated by the systematic decrease of MgO, Fe_2_O_3_^T^, TiO_2_, CaO, P_2_O_5_, and Sr with increasing SiO_2_, consistent with progressive removal of mafic silicates, Fe–Ti oxides, apatite, and plagioclase. Superimposed on these differentiation trends, variable K/Rb and Ba/Nb ratios, enrichment in Rb–Th–U–Pb, and non-closed-system CaO/Na_2_O–SiO_2_ relationships suggest crustal input during magma evolution^71–74^. Field and petrographic observations further support this interpretation, as mafic enclaves in the syn-orogenic granites (Fig. 2b, d) and complex plagioclase zoning (Fig. 4b) are compatible with magma interaction and disequilibrium crystallization. However, such enclaves may also represent cognate mafic aggregates, restitic residues, or xenolith fragments. Because isotopic or mineral-scale diffusion constraints are unavailable, they are regarded as supporting evidence for open-system magmatic processes rather than definitive proof of magma mixing. Geochemically, the syn-orogenic granites display wide scatter in trace-element ratios that would remain more constant under closed-system fractionation (e.g., highly variable K/Rb and Ba/Nb; Supplementary Table 3; Fig. 10a), indicating substantial crustal assimilation^5,69^. The monzogranites also exhibit evidence of assimilation coupled with fractional crystallization: their elevated Rb (up to 178 ppm) and non-constant K/Rb ratios fit an AFC trend^75^ rather than simple fractional crystallization (Fig. 10a). Both suites show declining CaO/Na_2_O with silica (Fig. 10b), further supporting an AFC-dominated evolution. Because no Sr-Nd-Hf isotope data are presented, AFC is discussed qualitatively and not modeled quantitatively.

Fractional crystallization of specific minerals controlled much of the differentiation. Plagioclase removal is indicated by linear declines in Sr and CaO with increasing SiO_2_ (Fig. 7b, h) and by co-variation of Zr and TiO_2_ (Fig. 10c), the latter also implicating accessory zircon and titanite crystallization. Consistently, the evolved monzogranites show deep negative Eu, Sr, and Ba anomalies (Fig. 8b) from extensive feldspar (plagioclase ± K-feldspar) extraction. The albite-rich (Ab_77.9–99.8_) composition (Supplementary Table 2) of plagioclase and the concentration of zircon and columbite-tantalite minerals (Supplementary Table 2) provide robust mineralogical evidence for the highly evolved nature of the monzogranite melts. Biotite compositions (Fe# = 0.41‒0.43) are consistent with Fe enrichment; however, their use as petrogenetic indicators should be treated with caution due to possible subsolidus re-equilibration (Fig. 5d). In contrast, the syn-orogenic granites have only a mild Eu anomaly (Fig. 8c), implying limited plagioclase fractionation. Simultaneous crystallization of ferromagnesian silicates is evidenced by decreasing MgO and Fe_2_O_3_ with increasing SiO_2_ (Fig. 7c, d), and early apatite and Fe-Ti oxide removal by the decline of P_2_O_5_ and TiO_2_ and corresponding P, Ti troughs in multi-element patterns (Figs. 7a and g and 8b and d). In contrast, increasing K_2_O and Rb at higher SiO_2_ (Fig. 7f, i) suggests late-stage K-feldspar accumulation in the monzogranite melts. These processes yielded increasingly Fe-enriched, differentiated magmas (FeO/MgO up to ~ 8; Laurent, et al.^76^ and produced pronounced LREE-over-HREE enrichment in both suites (La_N_/Lu_N_ up to ~ 20; Fig. 8a, c).

### Magma type and tectonic setting signature

The geochemical contrasts between the Abu Murrat granite suites reflect different tectonic origins. Granitic magmatism in the Eastern Desert evolved from subduction-related arcs to post-collisional intraplate settings^77,78^. Accordingly, the syn-orogenic granites correspond to arc-derived I-type intrusions, whereas the post-orogenic monzogranites represent post-orogenic A-type granites emplaced during extension^79^.

Discrimination diagrams further support the compositional contrast between the two granitoid suites. On the FeO^t^/(FeO^t^+MgO) vs. SiO_2_ diagram of Frost, et al.^67^, the syn-orogenic granites plot in the magnesian field, whereas the post-orogenic granites are mainly ferroan to transitional. The later have an average Fe# of approximately 0.8, although some samples lie close to the magnesian–ferroan boundary (Supplementary Table 3; Fig. 9b). This distribution highlights the contrast between comparatively hydrous, magnesian arc-related magmas and more evolved, Fe-enriched post-orogenic magmas. However, because oxygen fugacity was not independently quantified, redox state is not inferred from Fe-number alone. The post-orogenic granites also occupy alkali-calcic to alkalic fields in that diagram, unlike the calc-alkaline syn-orogenic granites (Fig. 9c). On an alumina saturation plot (A/NK vs. A/CNK^68^; Fig. 9d), the post-orogenic granite suite spans metaluminous to peraluminous compositions, whereas the syn-orogenic granites cluster in the metaluminous field, a difference reflecting the extreme degree of fractional crystallization in the post-orogenic suite.

**Fig. 11:**
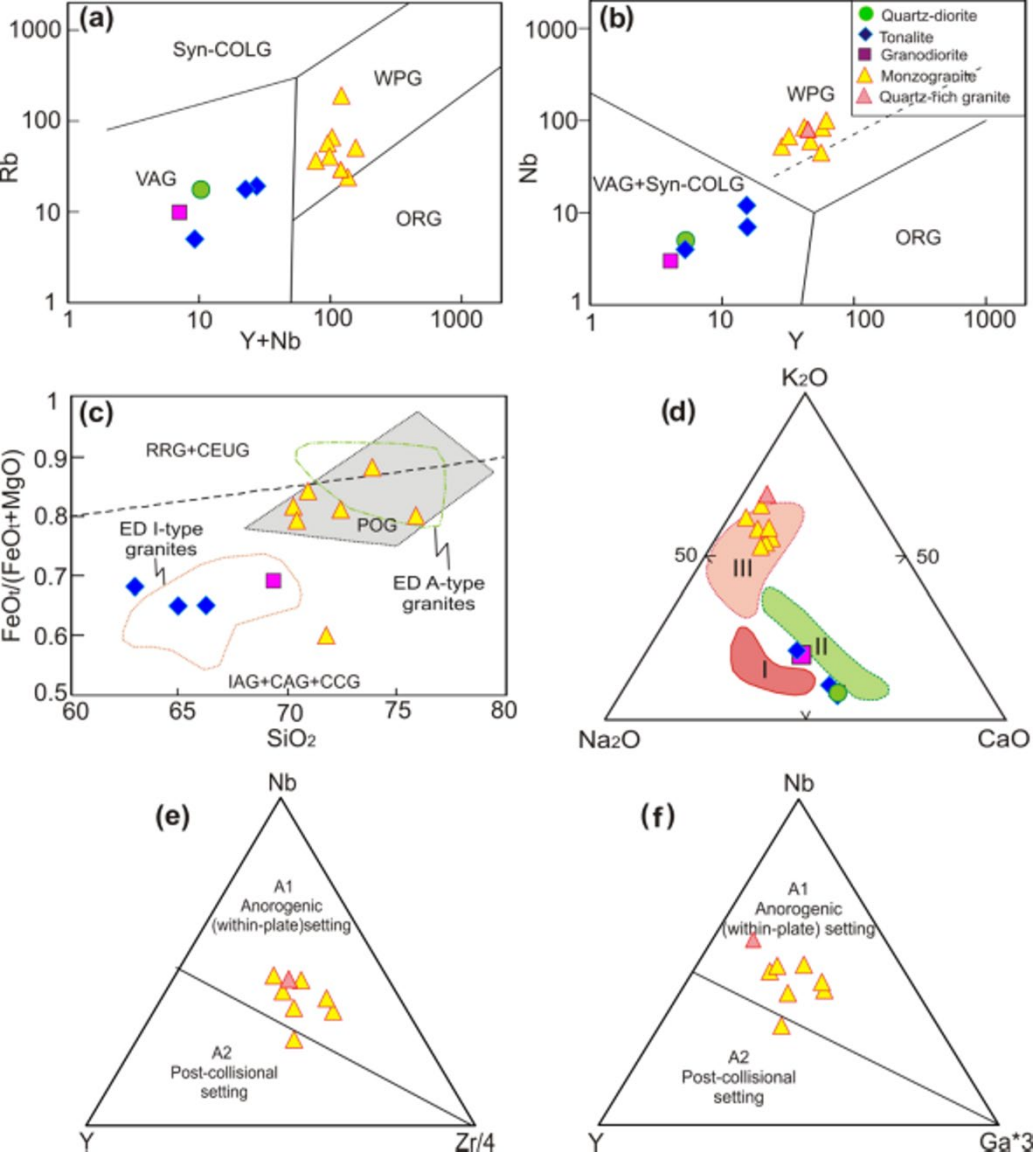
Tectonic discrimination diagrams. (**a**) Rb vs. Y + Nb and (**b**) Nb vs. Y^78^ with syn-orogenic granites plotting in volcanic-arc granite (VAG) field and post-orogenic granites in within-plate granite (WPG) field. (**c**) FeO^t^/(FeO^t^+MgO) vs. SiO_2_^68^ with fields for Eastern Desert A-type vs. I-type granites after Azer, et al.^5^, Farahat, et al.^24^. (**d**) Na_2_O–K_2_O–CaO ternary^49,57^ showing Egyptian granite groups I (old calc-alkaline), II (early phase young calc-alkaline), III (late phase young calc-alkaline). (**e**) Nb–Y–Zr/4 and (**f**) Nb–Y–3Ga ternary plots^82^ distinguishing A1-type vs. A2-type A-type granites. Younger monzogranites show A1-type affinity (within-plate, OIB-like source) in both diagrams, consistent with other Eastern Desert A-type granites, e.g., El-Bialy and Omar^62^.

The tectonic discrimination diagrams are consistent with a transition from subduction-related magmatism to post-orogenic intraplate magmatism, but they are interpreted in conjunction with the field relations and mineralogical evidence. On tectonic discrimination plots (Rb vs. Y + Nb and Nb vs. Y; Fig. 11a–b)^78^, the Abu Murrat syn-orogenic granites plot in the volcanic-arc granite field (VAG), supporting an arc or active continental margin origin, whereas the post-orogenic granite samples plot in the within-plate granite field (WPG), consistent with an intraplate A-type affinity. Similarly, the monzogranite suite overlaps post-orogenic granite fields, whereas the syn- to late orogenic suite plots with island-arc granites (Fig. 11c). In a K_2_O–Na_2_O–CaO ternary, the syn- to late orogenic granites cluster with subduction-related “Phase II” calc-alkaline intrusions (~ 635–620 Ma), whereas the post-orogenic cluster with late “Phase III” post-collisional granites (~ 610–600 Ma)^49,57^ (Fig. 11d). Y–Nb–Ga systematics suggest that the post-orogenic monzogranites predominantly exhibit A1-type A-granite characteristics, consistent with a mantle-influenced intraplate signature, while some samples interpret as A1-leaning transitional A-type granites 80 (Fig. 11e, f). This is consistent with their HFSE-rich, Sr- and P-poor chemistry (Supplementary Table 3; Fig. 8b) and with other Eastern Desert A1-type granites^62,79,81^. The monzogranites’ REE pattern (flat HREE, strong Eu anomaly; Fig. 8a) closely resembles those of analogous anorogenic granites in the region^5,18^. The geological and geochemical evidence as a whole strongly supports that the syn- to late orogenic granites formed in a compressional, subduction-related tectonic setting, whereas the post-orogenic granites were emplaced during post-collisional extensional tectonism.

### Geodynamic model

**Fig. 12:**
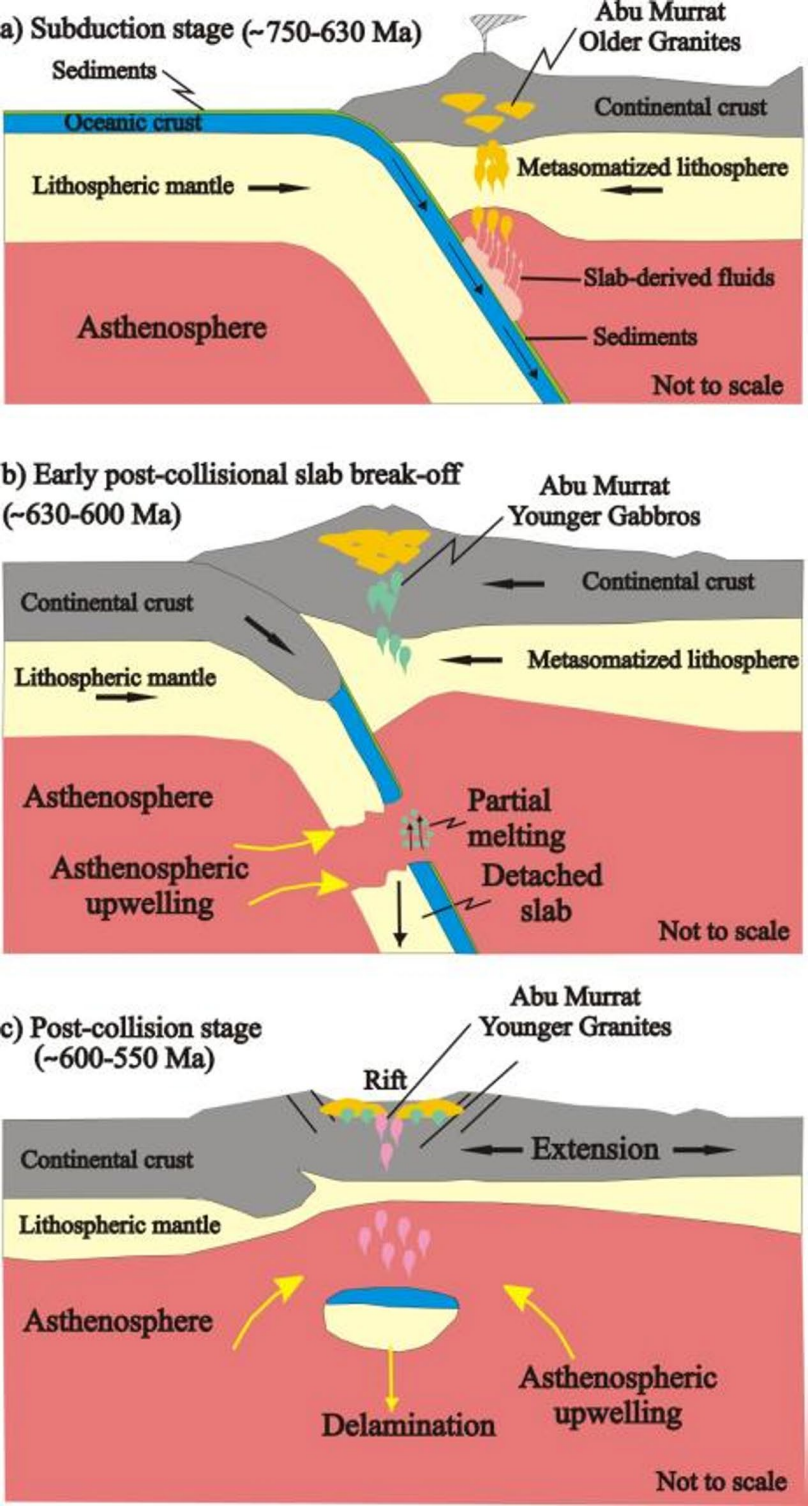
Schematic model for the emplacement of Abu Murrat granitoids through successive Pan-African tectonic stages (not to scale). (**a**) Compressional (subduction) stage: formation of syn-orogenic granites (I-type) in a volcanic arc setting by partial melting of mantle wedge and AFC of lower crust. (**b**) Slab break-off stage: detachment of subducted slab at end of collision triggers mantle upwelling and ferrobasaltic magmatism (post-orogenic gabbros) in a narrow slab window. (**c**) Extensional (post-collisional) stage: lithospheric delamination and asthenospheric upwelling cause metasomatism of lithosphere and partial melting of tonalitic lower crust, generating metaluminous to peraluminous A-type monzogranite magmas (post-orogenic granites) that intrude syn-orogenic crustal rocks. The timing of the tectonic stages is based on published geochronological constraints from the ANS and are not new dates from the present study.

We propose a plausible three-stage regional-to-local tectono-magmatic model for the Abu Murrat granitoids (Fig. 12). This model is based on regional geochronological constraints, cross-cutting field relationships, and comparison with nearby Eastern Desert plutonic suites, as no new U–Pb geochronological data were obtained in the present study. The proposed stages are as follows: (1) During the late syn-collisional (compressional) stage (~ 630–610 Ma, at the culmination of the Pan-African Orogeny), subduction of oceanic lithosphere beneath the northern Arabian–Nubian Shield (ANS) promoted hydrous melting of the mantle wedge and emplacement of calc-alkaline, I-type magmas at depth^2,12,83^. These mantle-derived basaltic to andesitic magmas likely accumulated near the crust–mantle boundary, transferring heat and driving partial melting and assimilation of lower-crustal rocks^84,85^. Through these coupled processes, the Abu Murrat quartz diorite–tonalite–granodiorite suite (syn-orogenic granitoids) can be explained as a continental-arc assemblage (Fig. 12a)^10,62^. Mafic enclaves and geochemical evidence for AFC in the syn-orogenic granites are consistent with this scenario^10,86^. (2) Following collision, slab detachment probably developed during the early post-collisional slab break-off stage (~ 630–600 Ma), with the main break-off event inferred near 600 Ma. This created a transient pathway for asthenospheric inflow and decompression melting beneath the thickened orogen^10,87^. Upwelling and decompression melting of metasomatized lithospheric mantle in this incipient extensional regime produced voluminous mafic (ferrobasaltic) magmas, represented locally by the Abu Murrat younger gabbros and comparable post-collisional gabbroic intrusions in the northern ANS (Fig. 12b)^26,88^. The emplacement of the Abu Murrat younger gabbros is consistent with the earliest stages of post-orogenic extension (Stage c), as documented for the northern ANS during late Ediacaran collapse^10,12^. (3) During the post-compressional (extensional) stage, crustal thinning and lithospheric delamination following slab removal enhanced asthenospheric upwelling beneath the region (Fig. 12c)^10^. Basaltic underplating and associated alkaline fluid fluxes are expected to fertilize the lower crust—raising alkali and HFSE budgets and priming it for later anatexis^10,89,90^. This interpretation aligns with Martin’s model for A-type granite genesis, where mantle-derived melts and CO_2_-H_2_O-rich fluids metasomatize (fertilize) the lower crust during extension, thereby facilitating generation of ferroan A-type granitoids^89^. High-temperature mantle input then enabled partial melting of pre-existing tonalitic lower crust, producing post-collisional, metaluminous to weakly peraluminous magmas that rose to form the monzogranites (A-type post-orogenic granites). These melts subsequently evolved by fractional crystallization, and locally, open-system differentiation, during ascent and storage, producing the observed evolved granitic compositions^69,91,92^. The post-orogenic granites intruded the syn-orogenic granitoids and the post-orogenic gabbros and are temporally younger (Fig. 12c), consistent with interpretations by several researchers (e.g., Azer, et al.^5^, El-Awady, et al. ^18^, El-Awady, et al. ^26^, Seddik, et al.^93^, who proposed that these rocks formed within an extensional regime linked to the post-collisional collapse of the Pan-African Orogeny. Therefore, the Abu Murrat granitic assemblage records a transition from subduction-related orogenesis (calc-alkaline I-type granitoids) to post-collisional extension (A-type granitoids), reflecting the latest Neoproterozoic evolution of the northern ANS from crustal thickening to delamination, asthenospheric upwelling, and incipient intracontinental rifting. Because isotopic data are not available here, this source interpretation is framed as the most consistent model rather than a uniquely demonstrated conclusion. The value of the Abu Murrat locality lies in preserving the older calc-alkaline granitoids, post-orogenic gabbros, and younger A-type granites in direct field association. This allows the regional subduction to extension transition to be evaluated at outcrop and suite scale.

## Conclusions

Neoproterozoic granitoids in the Abu Murrat district (northern Eastern Desert) reflect two distinct intrusive episodes. The older quartz diorite–tonalite–granodiorite suite is calc-alkaline, magnesian, and largely metaluminous (I-type), with arc-like trace-element features (e.g., low Nb–Y) and minor Eu anomalies, consistent with hydrous mantle-derived magmas evolved via assimilation–fractional crystallization during late Pan-African convergence. In contrast, younger monzogranites are alkali-rich and ferroan to transitional, metaluminous to mildly peraluminous, and enriched in HFSE–REE with pronounced negative Eu anomalies, reflecting extensive feldspar-dominated fractionation from dry, high-temperature A-type magmas. These younger intrusions are widely interpreted as post-collisional (~ 600 Ma) and related to late-orogenic extension, plausibly involving lithospheric root removal and asthenospheric upwelling that can promote lower-crustal melting.

Thus, the Abu Murrat granitoids record a transition from subduction-related arc magmatism to post-collisional intraplate magmatism in the northern Eastern Desert of Egypt. They exemplify how the waning stages of the Pan-African Orogeny in the Arabian–Nubian Shield produced a shift in granite character from I-type calc-alkaline plutons formed in a convergent tectonic regime to A-type granites generated during subsequent lithospheric extension.

## Supplementary Information

Below is the link to the electronic supplementary material.

Supplementary Material 1

Supplementary Material 2

## Data Availability

The datasets generated and analyzed during the current study are available in the Supplementary Information file.
