# Supplementary material for: Petrological and geochemical characterization of Abu Murrat syn-orogenic I-type and post-orogenic A-type neoproterozoic granitoids, North Eastern Desert, Egypt
Source: Sci Rep. 2026 Jul 15;16:22249. doi: 10.1038/s41598-026-61098-1 (PMC13372817; doi:10.1038/s41598-026-61098-1)
Supplement: Supplementary file 1 — Supplementary Material 1 [file 41598_2026_61098_MOESM1_ESM.pdf]

Supplementary Table 1. Modal composition of the studied granitic rocks.

Sample No.	Bd2	Bd39	Bd46	Bd44	Bd60	Bd85	Bd9	Bd50	Bd51	Bd61	Bd65	Bd68
Rock Unit	Older granites					Younger granites						
Petrology	Tonalite			Granodiorite	Qz-diorite	Monzogranite					Syenogranite	
Quartz vol.%	25	27.5	28.03	32.6	10.9	31.8	28	30	28	31	32	33
Plagioclase vol.%	50.2	51.7	55.7	50.05	62.5	52.4	33	31	31	30	20	15.5
K-feldspars vol.%	1.5	3.9	3.09	9.25	2.3	10.1	35	32	33.7	34	43	45
Mafic minerals vol.%	20.4	13.9	11.11	6.4	22.9	4.3	3	5.5	5.3	4	4	5.3
Accessory vol.%	2.9	3	2.07	1.7	1.4	1.4	1	1.5	2	1	1	1.2
Total	100	100	100	100	100	100	100	100	100	100	100	100

Supplementary Table 3. Major (wt%) and trace element (ppm) abundances of Abu Murrat granitic rocks.

Sample No.	Bd85	Bd7	Bd9	Bd50	Bd51	Bd61	Bd41	Bd13	Bd43	Bd2	Bd39	Bd46	Bd60	Bd44
Rock unit	Post-tectonic Granites									Syn-tectonic Granites				
Petrology	Monzogranite							Qz-rich granite	Microgr dike	Tonalite			Quartz diorite	Grano-diorite
SiO2	71.73	70.89	72.39	70.22	73.84	75.84	70.38	89.83	56.07	66.22	62.96	64.94	59.72	69.31
TiO2	0.13	0.12	0.52	0.31	0.18	0.13	0.10	0.07	1.71	0.51	0.45	0.66	0.66	0.44
Al2O3	14.74	14.34	13.18	14.14	14.08	12.00	15.00	4.80	15.62	15.16	13.43	15.43	17.14	15.48
Fe2O3	1.02	1.14	1.80	1.91	1.89	0.91	0.99	0.42	9.01	3.31	4.16	4.43	4.83	1.54
MnO	0.07	0.02	0.08	0.03	0.01	0.01	0.02	<0.01	0.18	0.10	0.07	0.08	0.07	0.04
MgO	0.62	0.20	0.39	0.40	0.24	0.21	0.24	0.12	2.06	1.60	1.75	2.15	1.73	0.62
CaO	1.47	1.52	1.23	0.72	0.84	1.16	0.56	0.24	6.51	3.98	7.33	5.60	7.19	4.12
Na2O	3.32	3.88	3.50	3.39	3.13	3.04	4.54	1.04	5.10	4.89	5.67	4.70	5.52	4.81
K2O	5.77	6.14	5.12	7.59	5.36	5.76	7.99	2.75	1.05	2.31	0.94	1.15	1.05	2.15
P2O5	0.12	0.05	0.24	0.10	0.03	0.03	0.03	0.04	0.92	0.21	0.14	0.26	0.23	0.35
LOI	0.64	1.28	1.75	0.89	0.78	0.75	1.02	0.56	1.43	1.34	2.93	1.00	2.04	1.52
Sum	99.63	99.58	100.20	99.70	100.38	99.84	100.87	99.87	99.66	99.63	99.83	100.40	100.18	100.38
A/CNK	1.03	0.91	0.97	0.94	1.13	0.90	0.88	0.94	0.73	0.85	0.57	0.81	0.74	0.87
A/NK	1.26	1.10	1.17	1.03	1.29	1.07	0.93	1.02	1.64	1.44	1.30	1.72	1.68	1.51
K2O/Na2O	1.74	1.58	1.46	2.24	1.71	1.89	1.76	2.64	0.21	0.47	0.17	0.24	0.19	0.45
FeOt/FeOt+MgO	0.62	0.85	0.82	0.83	0.89	0.81	0.80	0.78	0.81	0.67	0.70	0.67	0.74	0.71
K/Rb	391.13	345.818	316.89	389.02	249.83	576.074	849.62	837.42	1909.5	656.1	222.80	234.50	231.96	447.37
Trace elements														
As	24	21	20	23	17	23	24	21	20	15	27	22	18	16
Cs	0.43	0.18	0.16	0.35	0.39	0.19	0.08	0.09	0.07	0.15	0.01	0.32	0.14	0.07
Rb	122.47	147.39	134.13	161.97	178.10	83.00	78.07	27.26	4.56	29.23	35.02	40.71	37.58	39.90
Ba	256	690	601	1848	458	694	273	167	493	257	352	488	331	922
Th	15.16	13.01	13.14	15.86	22.75	14.51	14.83	10.24	0.43	4.98	2.26	5.31	4.38	4.13
U	1.98	2.42	2.76	3.86	5.88	3.63	2.91	1.31	0.12	1.33	1.11	1.32	1.15	1.32
Nb	78	94	49	56	79	63	42	75	47	12	4	7	5	3
La	11.88	13.83	23.10	66.14	52.71	14.57	9.71	4.08	42.78	9.73	4.96	15.95	9.95	13.79
Ce	29.90	26.97	54.63	137.88	119.81	27.57	21.61	8.28	110.95	23.95	11.34	37.45	19.80	25.92
Pb	7	9	6	11	10	6	8	5	2	6	4	7	4	5
Pr	3.91	3.63	6.99	15.20	15.92	3.82	2.97	0.83	15.91	2.79	1.37	5.13	2.36	2.52
Sr	123	236	370	204	77	210	90	39	551	348	576	537	883	388
Nd	17.00	12.27	29.74	65.23	63.32	11.94	9.32	3.16	71.49	10.68	5.67	22.02	9.58	9.88
Zr	239	217	270	231	245	286	271	208	1043	94	78	143	110	57
Hf	15.16	17.20	16.01	15.21	18.86	20.59	13.20	11.19	0.39	1.00	0.66	0.89	0.40	0.39
Sm	4.78	3.41	6.37	16.72	15.45	3.36	3.24	0.79	16.52	2.14	1.50	5.11	1.84	1.69
Eu	0.66	0.19	0.17	0.29	1.56	0.12	0.10	0.13	4.69	0.61	0.44	1.21	0.46	0.51
Gd	5.47	4.09	6.28	15.83	14.21	3.85	2.42	0.84	16.20	2.09	1.33	4.30	1.52	1.50
Tb	0.90	0.82	1.01	2.18	2.03	0.77	0.59	0.11	2.43	0.28	0.21	0.62	0.24	0.17
Dy	5.83	4.48	5.57	9.02	9.18	3.59	2.89	0.90	13.78	1.51	1.23	3.52	1.36	1.09
Y	57.63	61.71	28.36	46.81	41.98	32.15	56.34	44.85	60.14	18.37	12.25	17.55	13.31	14.07
Ho	1.13	1.03	1.09	1.90	1.50	0.77	0.57	0.21	2.58	0.35	0.24	0.61	0.25	0.19
Er	3.71	2.79	3.26	4.99	4.39	2.03	1.87	0.71	6.76	0.90	0.71	1.88	0.79	0.63
Tm	0.50	0.45	0.41	0.67	0.64	0.31	0.25	0.10	0.86	0.15	0.11	0.21	0.11	0.07
Yb	3.24	2.13	2.89	4.86	4.47	1.56	1.87	0.74	5.47	0.84	0.66	1.58	0.69	0.49
Lu	0.47	0.39	0.39	0.84	0.73	0.34	0.27	0.11	0.78	0.11	0.09	0.24	0.09	0.07
V	22	10	33	13	4	5	5	1	75	37	76	62	51	9
Co	82	49	51	54	41	54	80	65	61	43	82	63	35	25
Ni	6	2	30	4	2	4	3	1	16	14	13	21	17	3
Zn	32	12	49	25	7	16	13	6	149	71	36	64	57	23
Ga	11.08	13.55	14.63	13.02	21.12	11.85	14.70	4.78	22.48	12.08	12.56	17.75	20.77	15.50
Cu	4	1	4	3	2	1	1	<1	17	10	3	29	9	2
Cl	0.04	0.02	0.03	0.03	<0,01	<0,01	0.03	0.03	0.06	0.03	0.04	0.02	0.03	0.05
Sc	8	<1	2	<1	<1	<1	<1	<1	22	11	8	14	8	2
Mo	3	2	1	1	<1	1	1	1	<1	1	1	1	1	1
In	0.05	<0,01	<0,01	<0,01	0.04	0.03	<0,01	<0,01	0.07	<0,01	<0,01	<0,01	<0,01	<0,01
Ag	0.30	<0,02	0.09	0.09	0.36	0.25	<0,02	<0,02	0.61	0.15	0.04	0.05	0.05	<0,02
Au	0.05	0.02	0.02	0.02	0.06	0.04	<0,02	<0,02	0.08	0.03	<0,02	0.03	<0,02	<0,02
Ir	<0,02	<0,02	<0,02	<0,02	<0,02	<0,02	<0,02	<0,02	<0,02	<0,02	<0,02	<0,02	<0,02	<0,02
Pd	<0,02	<0,02	<0,02	<0,02	<0,02	<0,02	<0,02	<0,02	<0,02	<0,02	<0,02	<0,02	<0,02	<0,02
Pt	0.03	<0,02	<0,02	<0,02	<0,02	0.02	<0,02	<0,02	<0,02	<0,02	<0,02	<0,02	<0,02	<0,02
Rh	<0,02	<0,02	<0,02	<0,02	<0,02	<0,02	<0,02	<0,02	<0,02	<0,02	<0,02	<0,02	<0,02	<0,02
Ru	<0,02	<0,02	<0,02	<0,02	<0,02	<0,02	<0,02	<0,02	<0,02	<0,02	<0,02	<0,02	<0,02	<0,02
Sb	0.18	0.09	0.11	0.08	0.09	0.08	0.08	0.07	0.19	0.09	0.49	0.17	0.11	0.11

**Sn**	1.16	0.49	1.73	1.09	1.22	2.40	0.52	0.34	2.01	2.17	0.89	1.11	0.82	0.56
**Te**	<0,02	<0,02	<0,02	<0,02	<0,02	<0,02	<0,02	<0,02	<0,02	<0,02	<0,02	<0,02	<0,02	<0,02
**Sum REE**	89.4	76.5	141.9	341.8	305.9	74.6	57.69	20.99	311.20	56.14	29.84	99.83	49.04	58.53
**LaN/LuN**	2.62	3.68	6.15	8.17	7.50	4.45	3.73	4.00	5.69	9.18	5.72	6.90	11.47	20.42
**LaN/YbN**	2.49	4.41	5.43	9.24	8.01	6.34	3.53	2.83	5.31	7.86	5.10	6.86	9.79	19.14
**Eu/Eu***	0.39	0.16	0.08	0.05	0.32	0.10	0.11	0.49	0.88	0.88	0.95	0.79	0.84	0.98
**Ba/Nb**	3.28	7.34	12.27	33.00	5.80	11.02	6.50	2.23	10.49	21.42	88.00	69.71	66.20	307.33
**Norm**														
**Q**	26.61	22.20	29.90	20.03	33.06	34.16	14.66	72.82	10.05	19.87	14.31	20.57	9.56	25.18
**C**	0.65	0.00	0.22	0.00	1.67	0.00	0.00	0.00	0.00	0.00	0.00	0.00	0.00	0.00
**Or**	34.10	36.29	30.26	44.86	31.68	34.04	47.22	16.25	6.21	13.65	5.56	6.80	6.21	12.71
**Ab**	28.09	32.83	29.62	28.69	26.49	25.72	32.67	8.80	43.16	41.38	47.98	39.77	46.71	40.70
**An**	6.51	3.58	4.53	0.95	3.97	2.09	0.00	0.31	16.63	12.59	8.42	17.61	18.89	14.30
**Ac**	0.00	0.00	0.00	0.00	0.00	0.00	2.86	0.00	0.00	0.00	0.00	0.00	0.00	0.00
**Ns**	0.00	0.00	0.00	0.00	0.00	0.00	0.58	0.00	0.00	0.00	0.00	0.00	0.00	0.00
**Di**	0.00	1.08	0.00	0.79	0.00	1.13	1.29	0.30	3.43	3.42	9.40	5.05	9.30	1.93
**Wo**	0.00	0.80	0.00	0.00	0.00	0.67	0.27	0.00	0.00	0.00	5.70	0.00	0.55	0.00
**Hy**	1.54	0.00	0.97	0.63	0.60	0.00	0.00	0.16	3.54	2.40	0.00	3.01	0.00	0.65
**Ol**	0.00	0.00	0.00	0.00	0.00	0.00	0.00	0.00	0.00	0.00	0.00	0.00	0.00	0.00
**Il**	0.15	0.04	0.17	0.06	0.02	0.02	0.04	0.00	0.39	0.21	0.15	0.17	0.15	0.09
**Hm**	1.02	1.14	1.80	1.91	1.89	0.91	0.00	0.42	9.01	3.31	4.16	4.43	4.83	1.54
**Tn**	0.00	0.24	0.00	0.68	0.00	0.29	0.19	0.17	3.70	0.98	0.91	1.40	1.43	0.97
**Ru**	0.05	0.00	0.43	0.00	0.17	0.00	0.00	0.00	0.00	0.00	0.00	0.00	0.00	0.00
**Ap**	0.28	0.12	0.57	0.24	0.07	0.07	0.07	0.10	2.18	0.50	0.33	0.62	0.55	0.83
**Sum**	99.01	98.31	98.47	98.82	99.61	99.10	99.86	99.32	98.29	98.31	96.92	99.42	98.16	98.89
**TZr.sat.°C**	818	794	826	802	835	827	805	824	870	710	635	731	687	681
**Tmz.sat.°C**	722	685	754	815	862	691	655	632	690	647	502	666	593	665

Abbreviations: Qz-rich granite: Quartz-rich granite, microgranite dike.

Eu/Eu*= EuN/(SmN x GdN)*0.5

LaN, LuN, YbN values were normalized to chondritic compositions using the normalization constants of McDonough and Sun, 1995
